# Biomimetic nanoparticles with enhanced rapamycin delivery for autism spectrum disorder treatment via autophagy activation and oxidative stress modulation

**DOI:** 10.7150/thno.95614

**Published:** 2024-07-15

**Authors:** Chenlin Miao, Yizhe Shen, Yue Lang, Hui Li, Yan Gong, Yamei Liu, Huafei Li, Byron C. Jones, Fuxue Chen, Shini Feng

**Affiliations:** 1 School of Lifesciences, Shanghai University, 333 Nanchen Road, 200444, Shanghai, P.R.C.; 2 School of Environmental and Chemical Engineering, Shanghai University, 333 Nanchen Road, 200444, Shanghai, P.R.C.; 3 Department of Genetics, Genomics and Informatics, University of Tennessee Health Science Center, Memphis, USA.

**Keywords:** Autism spectrum disorder, Rapamycin, Blood-brain barrier, Autophagy, Oxidative stress

## Abstract

**Rationale:** Autism spectrum disorder (ASD) represents a complex neurodevelopmental condition lacking specific pharmacological interventions. Given the multifaced etiology of ASD, there exist no effective treatment for ASD. Rapamycin (RAPA) can activate autophagy by inhibiting the mTOR pathway and has exhibited promising effects in treating central nervous system disorders; however, its limited ability to cross the blood-brain barrier (BBB) has hindered its clinical efficacy, leading to substantial side effects.

**Methods:** To address this challenge, we designed a drug delivery system utilizing red blood cell membrane (CM) vesicles modified with SS31 peptides to enhance the brain penetration of RAPA for the treatment of autism.

**Results:** The fabricated SCM@RAPA nanoparticles, with an average diameter of 110 nm, exhibit rapid release of RAPA in a pathological environment characterized by oxidative stress. *In vitro* results demonstrate that SCM@RAPA effectively activate cellular autophagy, reduce intracellular ROS levels, improve mitochondrial function, thereby ameliorating neuronal damage. SS31 peptide modification significantly enhances the BBB penetration and rapid brain accumulation of SCM@RAPA. Notably, SCM@RAPA nanoparticles demonstrate the potential to ameliorate social deficits, improve cognitive function, and reverse neuronal impairments in valproic acid (VPA)-induced ASD models.

**Conclusions:** The therapeutic potential of SCM@RAPA in managing ASD signifies a paradigm shift in autism drug treatment, holding promise for clinical interventions in diverse neurological conditions.

## Introduction

Autism spectrum disorder (ASD) is a complex, pervasive and multifactorial neurodevelopmental disorder characterized by impairments in communication and social interaction, stereotyped and repetitive patterns of behavior and severe anxiety 1. A systematic review of epidemiological research estimates that the global prevalence of ASD in children is 1/36 (or 0.3%) over the past two decades 2. Additionally, due to the complex etiology, there is no single definitive treatment for all symptoms of ASD 3-7. mTOR, a conserved serine/threonine protein kinase in the phosphoinositide-3-kinase (PI3K) family, integrates various intracellular and extracellular signals, coordinating multiple cellular responses such as apoptosis, proliferation, and autophagy. Intact mTOR signaling is crucial for long-term synaptic plasticity and spatial learning 8. Studies indicate that approximately 8-10% of autism cases are due to abnormal mTOR signaling pathways 9, and up to 58% of autism susceptibility genes are directly or indirectly associated with mTOR signaling pathways 10. Furthermore, studies on layer V pyramidal neurons in the temporal lobe postmortem in individuals with idiopathic ASD have shown that aberrant mTOR pathway activity compromises autophagy, increasing dendritic spine density and decreasing developing dendritic spine pruning 11. Given that aberrant deregulation of the mTOR signaling system can contribute to ASD development, it is very likely that mTOR represents a prospective therapeutic target for ASD. Rapamycin (RAPA) functions as a specific inhibitor of the mTOR signaling pathway. It inhibits aberrant mTOR activation by blocking the binding of mTOR to its intracellular receptor protein and reducing the level of phosphorylation, thereby effectively boosting autophagy 12. A study conducted on Purkinje cell Tsc1 mutant mice indicates that RAPA can prevent pathological defects and ASD-like abnormalities 13. Besides, RAPA can promote autophagy and ameliorate symptoms in ASD model mice induced by valproic acid (VPA) by inhibiting mTOR activity 14. Therefore, RAPA exhibits potential value in treating ASD, suggesting promise for clinical investigation.

Although RAPA shows promise in treating autism, its hydrophobic nature presents challenges in clinical practice 15. The development of nano-delivery systems addresses conventional drug limitations, offering effective strategies for precise drug targeting and enhanced efficacy. Inspired by biological systems, researchers have turned to cellular membrane-mimetic nanocarriers for drug loading 16. Among these, red blood cells stand out due to their abundance in human blood, ease of extraction at scale, low immunogenicity, high biocompatibility, and biodegradability. Studies indicate that nanoparticles modified with red blood cell membranes (CM) effectively reduce recognition and ingestion by macrophages in the body, significantly extending circulation time and improving the biocompatibility of nanomaterials 17. Despite these advantages, limitations in active brain targeting and blood-brain barrier (BBB) penetration restrict the application of red blood cell membrane nanocarriers in treating central nervous system diseases. SS31 peptide (D-Arg-2'6'- dimethyltyrosine-Lys-Phe-NH_2_) is a mitochondrion-targeting peptide that is easily absorbed by endothelial cells and neurons due to its alternating aromatic cation residues, thus possessing excellent BBB penetration capabilities. Sun et al. modified SS31 peptide on the surface of nanovesicle, achieving precise drug delivery to central neurons, delayed the pathological process of traumatic brain injury 18. Furthermore, SS31 peptide has been shown to exhibit positive targeting effects in central nervous system diseases such as Parkinson's and Alzheimer's diseases 19-24.

**Figure 1:**
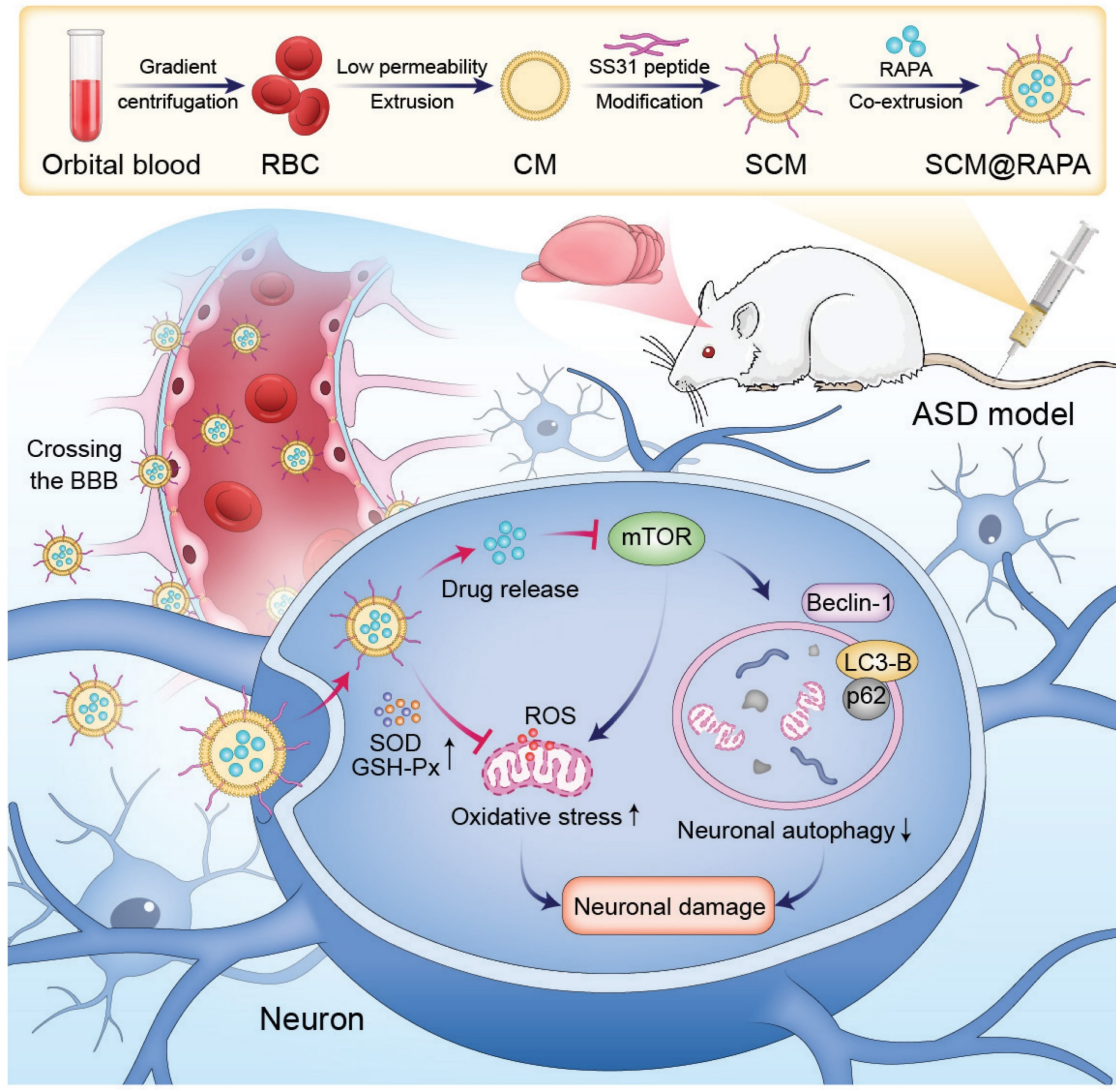
Schematic representation illustrating the preparation of blood-brain barrier (BBB)-penetrating SCM@RAPA and its therapeutic effects in ASD. SCM@RAPA NPs efficiently traverse the BBB and release RAPA in response to the oxidative stress pathological environment, thereby effectively enhancing neuronal autophagy, reducing intracellular ROS levels, and ameliorating neuronal damage for ASD treatment.

Here, the autophagy activator RAPA was encapsulated within red blood cell membrane vesicles and modified their surface with SS31 peptide, creating SCM@RAPA biomimetic nanoparticles (NPs) capable of targeting brain tissues for possible autism therapy (Figure 1). SCM@RAPA NPs can cross the BBB, accumulate significantly in the brain, and efficiently release drugs in response to the oxidative stress pathological environment. Furthermore, SCM@RAPA NPs effectively enhance neuronal autophagy, reduce intracellular ROS levels, improve mitochondrial functional impairments and ameliorate neuronal damage. Accordingly, SCM@RAPA NPs demonstrate the potential to enhance social abilities, decrease anxiety levels, ameliorate repetitive behaviors, and improve learning and memory in ASD models by activating autophagy and alleviating oxidative stress. Altogether, our results indicate that the SCM@RAPA NPs are a potential therapeutic candidate for precise drug treatment of autism.

## Methods

### Materials

RAPA (purity≥99%) and VPA (purity≥99%) was purchased from Sigma-Aldrich (St. Louis, MO, USA). DSPE-PEG-SS31 peptide was purchased from Xi'an Ruixi Biological Technology (Xi'an, China). Bicinchoninic acid (BCA) protein-assay kit, Sodium dodecyl sulphate-polyacrylamide gel electrophoresis (SDS-PAGE) gel-preparation kit, 2,7-dichlorodihydrofluorescein diacetate (DCFH-DA) probe, DAPI, and JC-1 were obtained from Beyotime Biotechnology (Shanghai, China). Cell counting kit-8 assay (CCK-8) was provided by Dojindo (Kumammoto, Japan). The antibodies were purchased from Cell Signaling Technology (CST, USA). All reagents were commercially available and used without further purification.

### Cells and animals

Human neuroblastoma cell line (SH-SY5Y) and mouse brain capillary endothelial cell line (bEnd.3) were obtained from Shanghai Dafei Biotechnology (Shanghai, China). Cells were cultured in DMEM containing 10% FBS, 1% penicillin/streptomycin at 37 °C and 5% CO_2_. Male and female Sprague-Dawley (SD) rats, weighing 250-350 g, were purchased from the Experimental Animal Center of the Chinese Academy of Sciences (Shanghai, China). All rats were housed individually in pathogen-free (SPF) conditions with controlled temperature of 23 ± 2 °C, humidity of 55% ± 5%, and a 12 h light-dark cycle. All experimental procedures were carried out in accordance with the guidelines in “the Animal Management Regulations” of the Ministry of Health of the People's Republic of China and were approved by the Animal Ethics Committee of Shanghai University (ECSHU-2020-030).

### Preparation of SCM@RAPA NPs

Erythrocytes were collected and isolated from the rat orbital venous plexus at 5000 rpm for 10 min and washed several times with pre-chilled 1×PBS until the supernatant was clarified. The obtained erythrocyte precipitate was lysed in hypotonic PBS (25%) for 4 h (shaking every 0.5 h) to facilitate content release. Subsequently, the supernatant was discarded by centrifugation with PBS (25%) at 8000 rpm for 10 min several times until the solution was colorless and the pink precipitate was collected as CM.

SCM@RAPA NPs were synthesized using the nano extrusion technique. DSPE-PEG-SS31 was introduced into the CM solution, which underwent sonication with a cell disruptor (190 W, 3 s on, 1 s off) using an ice bath probe for 10 min. The mixture was then incubated at 37 °C for 1 h, resulting in DSPE-PEG-SS31-CM (SCM). The ligand-modified drug was mixed with RAPA and extruded through polycarbonate membranes of varying pore sizes (800-100 nm) for 21 cycles. To remove any residual unbound ligands and drugs, the system was centrifuged at 12,000 rpm for 10 min and washed with PBS, yielding the SCM@RAPA NPs. Similarly, SCM@Cy5.5 NPs were prepared following the aforementioned protocol, wherein RAPA was replaced with Cy5.5.

### Characterization of SCM@RAPA NPs

The size distribution and zeta potential of various NP formulations were observed with a dynamic light scattering (DLS) analyzer (Nano-Brock 90Plus, USA). The morphological structure of SCM@RAPA NPs was visualized by uranyl acetate staining using a transmission electron microscope (TEM) (Hitachi HT7700, Japan). The surface protein content of SCM@RAPA NPs was determined by sodium dodecyl sulfate-polyacrylamide gel electrophoresis (SDS-PAGE) at 120 v for 1.5 h. The resulting gels were stained in Komasaki blue solution. Visualization was performed by overnight decolorization with a gel electrophoresis image system (Tanon, China). Fourier transform infrared (FT-IR) spectra of dried samples were collected using AVATAR 370 FT-IR (Nicolet, USA). X-ray photoelectron spectroscopy (XPS) measurements were performed using an Axis Ultra DLD X-ray photoelectron spectrometer (Kratos, UK). UV-Vis-NIR absorption spectra were recorded by Cary 5000 spectrophotometer (Agilent, USA).

### *In vitro* drug release analysis

The release of RAPA from SCM@RAPA NPs was evaluated using dialysis bags (MWCO 3000) submerged in 20 mL of 0.5% Tween-80 solution, with or without H_2_O_2_ (1 mM), at 37 °C with constant agitation (150 rpm). At specified intervals, 1 mL of the medium was withdrawn and replaced with an equal volume of fresh medium. The quantity of RAPA released was measured at 278 nm using a UV-visible spectrophotometer.

### Hemolysis assay

Rat venous blood cells were collected and washed three times with PBS solution without calcium and magnesium, followed by centrifugation at 1500 rpm to clarify the supernatant. The purified erythrocytes were resuspended in PBS to prepare a 2% erythrocyte suspension and stored at 4 °C for future use. Subsequently, 0.2 mL of the suspension was mixed with 0.2 mL of water, PBS, CM@RAPA, or SCM@RAPA and incubate for 3 h in a constant temperature water bath at 37 °C. Following incubation, all samples were centrifuged at 12,000 rpm for 10 min. 100 μl of the supernatant was aspirated into a 96-well plate with double distilled water and PBS as positive and negative controls, respectively. The absorbance value at 540 nm was measured via an automated microplate reader. The hemolysis rate (HR) was calculated according to the following equation:

HR (%) = 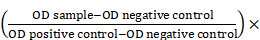 100%

### Cellular uptake

SH-SY5Y cells were seeded at a density of 1×10^5^ cells/well in confocal dishes and allowed to incubate overnight at 37 °C to ensure adherence. Hydrophilic fluorescent dye Cy5.5 was encapsulated into SCM NPs following the previously described method. Subsequently, free Cy5.5, CM@Cy5.5 and SCM@Cy5.5 were added to the dish separately and incubated with the cells for 4 h at 37 °C. Following this incubation period, cells underwent a thorough wash with PBS, followed by fixation with 4% paraformaldehyde solution for 20 min. Subsequently, nuclei were stained with DAPI, and fluorescence was examined via confocal laser scanning microscopy (CLSM). In a parallel setup, 3×10^5^ SH-SY5Y cells were cultured in 6-well plates for 24 h and then incubated with free Cy5.5, CM@Cy5.5 and SCM@Cy5.5 at 37 °C. Following another 4 h of incubation, cells were harvested by trypsin separation and resuspended with cold PBS. Flow cytometry assays were then conducted to assess the cellular uptake of the nanoparticles.

### Transportation across the *in vitro* BBB

To simulate the BBB *in vitro*, bEnd.3 cells were seeded in the upper chamber of transwell plates (pore size: 0.4 μm) to establish a BBB model for evaluating nanoparticle permeability. Once a dense monolayer of bEnd.3 cells formed, SH-SY5Y cells (1×10^5^) were seeded in the lower chamber and cultured for 24 h. Subsequently, Cy5.5, CM@Cy5.5 and SCM@Cy5.5 were added to the upper chamber after dilution with culture medium. After 4 h of incubation, the mean fluorescence intensity of SH-SY5Y cells was assessed by CLSM with an excitation wavelength of 690 nm or BD Calibur flow cytometry (BD Co., USA).

### *In vitro* cytotoxicity assay

A CCK-8 assay kit was used to detect cytotoxicity after treatment with NPs. SH-SY5Y cells and bEnd.3 were placed in 96-well plates at a ratio of 1×10^4^ cells/well and grown overnight at 37 °C. Cells were then exposed to SCM NPs without drug loading at concentrations of 200, 100, 50, 20, 10, 5, and 2.5 µg/mL for 24 h at 37 °C. Cells were lightly rinsed twice with pre-warmed PBS. Afterwards, each well was replaced with 100 µL of fresh medium containing 10% CCK-8 solution and incubated for 2 h. The absorbance was measured at 450 nm using an automated microplate reader.

The protective ability of SCM@RAPA NPs against VPA-induced cytotoxicity was assessed by the CCK-8 assay. SH-SY5Y cells were seeded in 96-well plates with approximately 1×10^4^ cells/well. Sample solutions containing VPA at a concentration of 20 mM and various NPs (RAPA, CM@RAPA and SCM@RAPA with the same RAPA concentration) were co-cultured with the cells for 24 h. Finally, cell viability was assayed separately using the CCK-8 assay kit.

### Intracellular ROS detection

The ROS scavenging ability of SCM@RAPA NPs in cells was determined using a DCFH-DA probe-based ROS assay kit. Free RAPA, CM@RAPA and SCM@RAPA NPs were diluted in cell culture medium and co-cultured with cells containing 20 mM VPA for 24 h at 37 °C. Subsequently, cells were labeled with DCFH-DA in serum-free medium for 30 min at 37 °C and 5% CO_2_ in an incubator. Following incubation, cells were washed three times with PBS, fixed with 4% paraformaldehyde solution for 20 min, and stained with DAPI for nucleus visualization. Fluorescence imaging was conducted via CLSM, and quantitative fluorescence intensity analysis was performed using flow cytometry, exciting at 488 nm in the FITC channel.

### Detection of mitochondrial membrane potential

Mitochondrial membrane potential (MMP) was stained with the JC-1 fluorescent probe following the protocol of manufacturer's instructions. Briefly, SH-SY5Y cells with VPA (20 mM) were incubated with free RAPA, CM@RAPA and SCM@RAPA NPs for 24 h, respectively. 1×10^6^ cells were resuspended in 10 mg/mL JC-1 and incubated for 20 min at 37 °C in the dark. Next, cells were washed three times with 1×PBS and aggregates were measured by flow cytometry at 490 nm excitation and monomers were measured at 530 nm emission. Fluorescence images were recorded by CLSM.

### Western blotting analysis

SH-SY5Y cells were treated with RAPA, CM@RAPA or SCM@RAPA in the presence of VPA, then harvested and resuspended in radioimmunoprecipitation assay (RIPA) buffer containing 10% phenyl methane sulfonyl fluoride (PMSF) to prepare the cell lysate. After incubation on ice for 20 min, the supernatant was collected by centrifugation (14,000 ×g, 30 min) at 4 °C. Protein concentration was determined using the BCA Protein Assay Kit. The same amount of proteins were run in 12% sodium dodecyl sulfate-polyacrylamide gel electrophoresis (SDS-PAGE) and then transferred to polyvinylidene difluoride (PVDF) membranes. Non-specific conjugates were incubated in 5% skim milk in TBST for 1 h at room temperature and then immunoblotted overnight at 4 °C with the following primary antibodies: LC3B (1:1000 dilution, CST), p62 (1:1000 dilution, CST), Beclin-1 (1:1000 dilution, CST) and glyceraldehyde-3-phosphate dehydrogenase (GAPDH) (1:1000 dilution Abcam). Afterwards, the blots were rinsed and incubated with the appropriate horseradish peroxidase (HRP)-conjugated secondary antibody (1:1000 dilution, Abcam) for 1 h at room temperature. The target protein was quantified using ImageJ.

### Detection of intracellular SOD and GSH-Px levels

SH-SY5Y Cells at a density of 1×10^5^ cells/well were plated overnight and added to RAPA, CM@RAPA or SCM@RAPA containing VPA (20 mM) and incubated at 37 °C for 24 h. Then, the cells were collected by centrifugation and lysed by freeze-thaw with liquid nitrogen and 37 °C water bath. Superoxide scavenging activity was measured by standard Superoxide Dismutase (SOD) assay kits and Glutathione peroxidase (GSH-Px) assay kits according to the manufacturer's protocol. Intracellular SOD activity was determined by WST-1 method. The protein concentration of the sample was determined by the BCA method, then 20 μL of enzyme dilution solution and enzyme working solution, and 200 μL of substrate application solution were added sequentially. The sample was incubated in a water bath at 37 °C for 30 min. The absorbance of the supernatant was measured by an enzyme meter at 450 nm, and the results were expressed in U mg/protein. The procedure of intracellular GSH-Px assay was as followed: first, the protein concentration of the sample was determined by BCA method. Subsequently, 1 mmol/L GSH was added sequentially, and reagent I and reagent II application solution were mixed with each reagent. The mixture was then reacted in a water bath at 37 ℃ for 5 min. Next, the samples were centrifuged at 3500 rpm/min for 10 min, and then 1 mL supernatant was taken from each group for color reaction. Following this, 1 mL of reagent III application solution, 0.25 mL of reagent IV application solution and 0.05 mL of reagent V application solution was added orderly, mixed well, and placed at room temperature for 15 min. Experiments were established with blank and standard tubes, and 1 mL of GSH standard solution or 20 μmol GSH standard solution instead of supernatant. The absorbance at 412 nm was detected and the intracellular GSH-Px activity was calculated.

### Brain targeting of SCM@RAPA NPs

The ability of the fabricated NPs to traverse the BBB and target brain was analyzed by a real-time imaging system 25. 1,1'-dioctadecyl-3,3,3',3'-tetramethylindodicarbocyanine,4-chlorobenzenesulfonate salt (DiD), served as fluorescence tracer, was incorporated into SS31-CM vesicles following the aforementioned protocol, while CM@DiD NPs were utilized as control counterparts. Both NPs were injected intravenously into male SD rats (PN35, 100g). Subsequently, the rats were placed in a chamber and imaged at 0.5, 2, 4, 6, 8 and 10 h post-injection. Fluorescence signals of the whole body were recorded with the IVIS Lumina II imaging system.

### Construction and treatment of ASD rat model

Female rats were mated with males at a ratio of 2:1 at 17:00. The following morning, vaginal obstruction was examined in female rats, marking embryonic day 0.5 (E0.5). Subsequently, these rats were individually housed in separate cages. On the 12.5^th^ day, pregnant rats in the experimental group received an intraperitoneal injection of VPA (500 mg/kg in 0.85% normal saline), while the control group received an equivalent volume of normal saline. Male offspring were retained for subsequent experiments. The offspring's birth day was recorded as PN0 (Postnatal, PN), and upon reaching PN35 (n = 6 per group) in the ASD rat model, RAPA, CM@RAPA, and SCM@RAPA (RAPA equivalent dose = 5 mg/kg) were administered daily via the tail vein for a 14-day treatment period. Behavioral tests were conducted three days after the commencement of the treatment.

### Three-chamber social test

The Three-chamber social test was used to measure social behavior and preference for novelty in rats. The subjects were introduced to three interconnected social boxes and allowed 10 min of free movement within the environment (designated as boxes A, B, and C) to acclimate. During the initial phase, a cage housing stranger 1 (a rat of the same strain and age as the test subject) was placed in box A, while an empty cage occupied box C. The movement of the subject rat was observed over the 10-min period. Subsequently, in the second phase, a cage containing stranger 2 (another rat of the same strain and age as the test subject) was positioned in box C, and the activity of the test rat was monitored for 10 min. Ethovision software was utilized to automatically calculate time spend in each chamber in the first and second stage.

### Open field test

ASD model rats treated with RAPA, CM@RAPA, or SCM@RAPA (RAPA equivalent dose = 5 mg/kg) were placed in an open field box for 10 min to acclimatize to the box environment, respectively. During the following 10 min, the total distance travelled and the time spent in the central area were recorded to test the anxiety and exploratory behavior of ASD rats.

### Elevated plus maze test

The elevated plus maze consists of two open arms, two closed arms and a common central platform 40 cm above the floor. The abnormal behavior of the experimental rats in the open arms may reflect their increased anxiety, mainly in the form of a significant decrease in the number of times the rats entered the open arms and the proportion of time they spent in the open arms. All experimental rats were gently introduced to the central area, facing the open arm directly, initiating a 10-min recording of their exploration within the elevated plus maze. The experiment was conducted in a quiet and unattended environment throughout.

### Y maze test

The Y maze apparatus (42 cm×15 cm×14 cm) was used to study the repetitive behavior of ASD rats. The Y maze device consisted of three identical arms. Rats were placed on the starting arm of the Y maze and allowed to randomly explore for 10 min, with their spontaneous alternation rate recorded as an indicator of repetitive behavior.

### Morris water maze behavioral test

The Morris water maze (MWM) was used to evaluate the spatial memory and learning ability of ASD rats. The system comprises a water-filled pool measuring 120 cm in diameter and 50 cm in height, housing an escape platform positioned beneath the water surface. The water temperature is maintained at 24 °C, with a depth of 18.5 cm equally divided into four quadrants, each serving as an entry point. In the most central area of the target quadrant, a circular platform with a 10 cm diameter sits 1 cm below the water surface. An installed camera captures the movements of the rat while connected to a computer for precise data collection and analysis. The MWM experiment was set for two phases: a training period and an exploratory period. The training spanned five days, with the rat introduced into the water from different quadrants daily. Each trial had a time limit of 60 s. If the rat failed to locate the platform within this time, it was guided to the platform and allowed to stay there for 20 s to aid in remembering its location. The exploratory phase occurred on the 6^th^ day. The platform was removed from the pool to assess the rat's memory of the platform's spatial location. The target quadrant, serving as the water inlet, was used for the experimental duration of 60 s. Latency to locate the simulated platform for the first time and the ratio of time spent in the target quadrant to the total swimming duration were recorded. The rat's swimming path and escape latencies were tracked using an image tracking system, and the data were analyzed using software (Ethovision XT, Panasonic, Japan).

### Immunoblotting of histones

After completion of the pharmacological and behavioral studies, ASD model rats treated with RAPA, CM@RAPA, or SCM@RAPA (RAPA equivalent dose = 5 mg/kg) were euthanized, and the hippocampus was isolated from the brain, supplemented with protease inhibitors at a rate of 1:100, and then transferred to the tissue protein extraction reagent for fragmentation to extract the proteins. Western blot experiments were performed as described in 2.12.

### TUNEL staining assay

TUNEL staining was performed using the TUNEL Apoptosis Analysis Kit, following the manufacturer's instructions. Firstly, hippocampus sections were deparaffinized by soaking in xylene, washed in gradient alcohol for 3 min, and rinsed with PBS solution for 5 min, followed by the addition of Proteinase K (20 μg/mL) dropwise to the tissue sections, and then digested for 20 min at 37 °C. Sample sections were incubated in 0.1% Triton X-100 solution at room temperature for 10-30 min. The samples were washed with PBS solution and incubated with TUNEL reaction mixture containing terminal deoxynucleotidyl transferase (TdT) and nucleotides for 2 h. At the end of the experiment, the nuclei of the cells were stained with DAPI staining solution, and the TUNEL-positive cells were observed under the fluorescence microscope and photographed for recording.

### Nissl staining assay

After routine dewaxing treatment, the tissue sections were placed in gradient alcohol for dehydration. The dehydrated tissue slices were then transferred to an oven at a temperature of 60°C, and then treated with 1% toluidine blue dye solution for 30 min. Unbound dyes were washed off with distilled water, and dehydrated in 70%, 80%, 95%, and 100% ethanol in sequence. Then, xylene was used for transparency. Finally, neutral gum was used to seal the slices and observed and photographed under a microscope.

### H&E staining assay

After the rats were euthanized, the heart, liver, lung, spleen and kidney tissues were taken out and washed with PBS to remove blood stains, and the tissues were immersed in 4% paraformaldehyde and fixed in a shaking bed for 24 h. The fixed tissues were then embedded in paraffin wax, and then sliced by paraffin slicer. The sections were deparaffinized with xylene and then dehydrated in gradient alcohol for 5 min each time, and then submerged in hematoxylin staining solution for 5 min and washed under running water for 15 min. Stained with eosin for another 5 min and wash off any excess dye on the surface with running water. The sections were dehydrated in 95% and 100% ethanol for 5 min each, and then soaked in xylene for 5 min until transparent. Finally, the sections were sealed with neutral resin, observed under a microscope and photographed.

### Single-cell RNA sequencing

Brain tissues were collected to extract total RNA tissue for building cDNA libraries and for sequencing. The official 10× genomic software CellRanger (v 5.0.0) was used for data quality statistics of raw data and cell quality control with the reference genome of Rat (https://ftp.ensembl.org/pub/release-98/fasta/rattus_norvegicus/). A unique molecular identifier (UMI) count matrix was processed and criteria were developed to remove low quality cells and possible multiple captures. After quality control, principal component analysis (PCA) was performed using the RunPCA function in Seurat to reduce dimensionality and clustering 26. To further identify cell types, the FindAllMarkers function was used to identify genes from different clusters. Differentially expressed genes (DEGs) were selected using the FindMarkers function (test.use = presto). P.adj < 0.05 and fold change > 1.2 were set as thresholds for significant differential expression. GO enrichment analysis of DEGs was performed using R based on hypergeometric distribution.

### Statistical analyses

All data were expressed as means ± standard errors of the mean (SEM) of at least three independent experiments performed on different days. Differences were considered statistically significant when the P-value was less than 0.05. ANOVA and Student's t-test statistical analyses were performed using the GraphPad Prism software unless otherwise specified.

## Results and Discussion

### Preparation and characterization of SCM@RAPA

**Figure 2:**
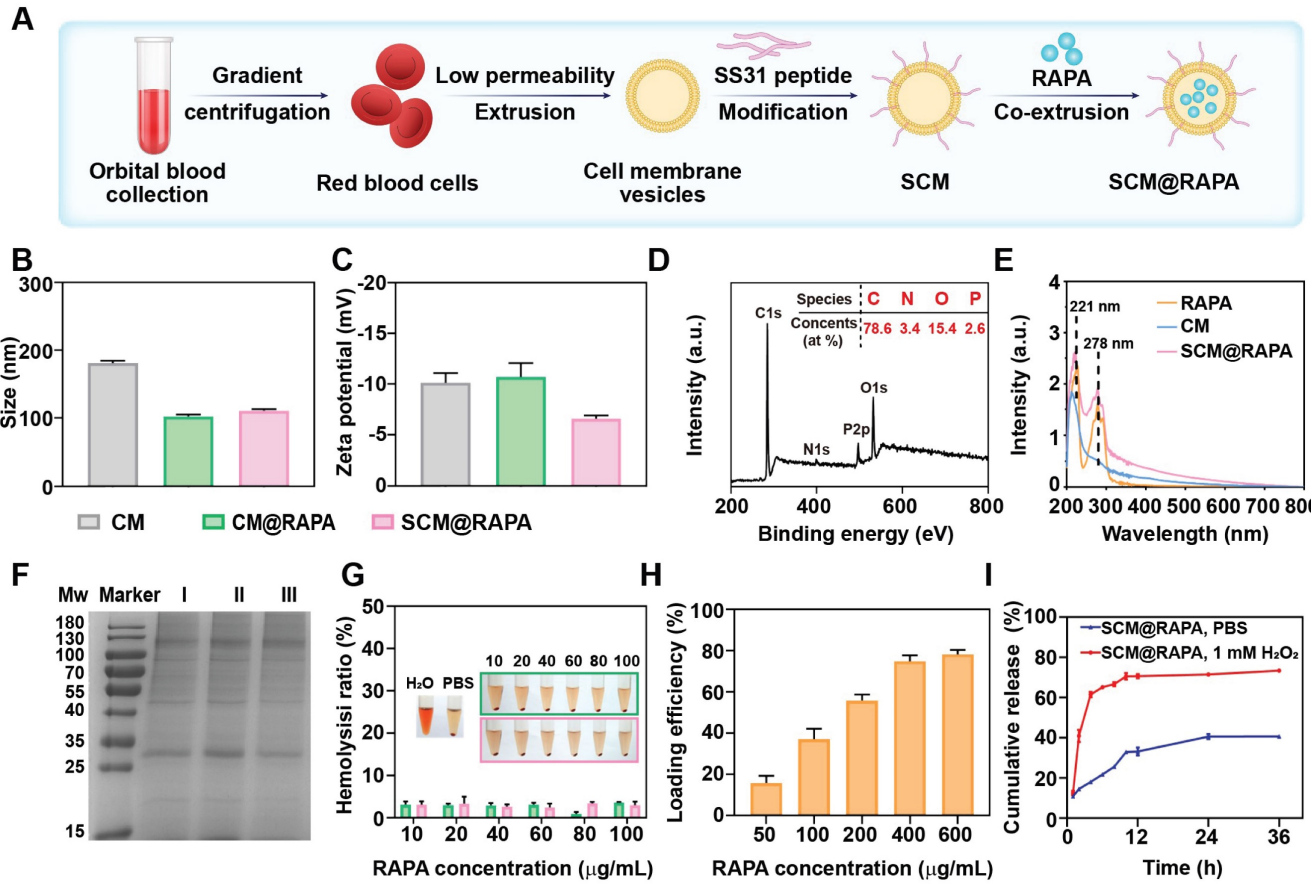
Characterization of SCM@RAPA. (A) Schematic diagram for preparation of SCM@RAPA. (B, C) DLS measurement of size and zeta potential of CM, CM@RAPA, and SCM@RAPA, respectively. (D) Survey XPS spectrum of CM@RAPA. (E) UV absorption spectrum of RAPA, CM and SCM@RAPA. (F) SDS-PAGE protein analysis of I CM, II CM@RAPA, and III SCM@RAPA. (G) Hemolysis quantification of red blood cell incubated with deionized water, PBS, CM@RAPA, and SCM@RAPA. (H) Encapsulation efficiency of RAPA with initial concentrations of 50-600 μg/mL. (I) Drug release in PBS (pH = 7.4) and H_2_O_2_ (1 mM) as measured by UV absorption.

SCM@RAPA nanoparticles (NPs), composed of RAPA encapsulated within an SS31-CM membrane shell, were synthesized via mechanical extrusion (Figure 2A). The SCM@RAPA NPs exhibited a spherical structure, averaging 100 nm in size with a 10 nm shell thickness, consistent with reported erythrocyte membrane dimensions 27 (Figure S1). Dynamic light scattering revealed a minor size increase from 102.4 nm to 110.8 nm after modification of cell membrane by SS31 peptides (Figure 2B). Furthermore, the CM@RAPA exhibited a surface potential similar to that of the erythrocyte membrane 28. Nevertheless, the surface charge negativity of SCM@RAPA diminished significantly in comparison to CM@RAPA (Figure 2C). This decline was attributed to the positively charged SS31 peptide countering a segment of the negative surface charge of the cell membrane, hence indirectly corroborating the successful integration of the SS31 peptide 29. The X-ray photoelectron spectroscopy spectrum revealed the presence of four notable peaks within the SCM@RAPA NPs, corresponding to C 1s at 284 eV, N 1s at 399 eV, P 2p at 510 eV, and O 1s at 531 eV (Figure 2D). The respective elemental composition was 78.61 at%, 3.4 at%, 15.4 at%, and 2.58 at%, with the phosphorus element originating from the erythrocyte membrane. Moreover, CM and RAPA exhibited characteristic UV-Vis absorption peaks at 221 nm and 278 nm, respectively (Figure 2E). The presence of these characteristic peaks on the spectrum of SCM@RAPA NPs further substantiated their successful construction. SDS-PAGE analysis showed membrane proteins in SCM@RAPA, resembling red blood cell-derived vesicles and confirming complete retention of membrane protein components (Figure 2F). Hemolysis assay and cell viability assessments on SH-SY5Y and bEnd.3 cells both affirming the excellent biocompatibility of SCM NPs (Figure 2G, S2). Figure 2H demonstrated that RAPA encapsulation efficiency reached 74.8% at RAPA concentration of 400 μg/mL. In addition, the drug loading capacity was calculated to be 195 μg/mg. To investigate RAPA release from the NPs, SCM@RAPA were incubated with or without H_2_O_2_ to simulate intracellular ROS conditions under physiological and pathological circumstances. In the presence of H_2_O_2_, the release of RAPA from SCM@RAPA NPs reached to nearly 70% at 12 h, indicating excellent ROS-responsive release characteristics (Figure 2I).

### Enhanced BBB Permeability of SCM@Cy5.5 *in vitro*

**Figure 3:**
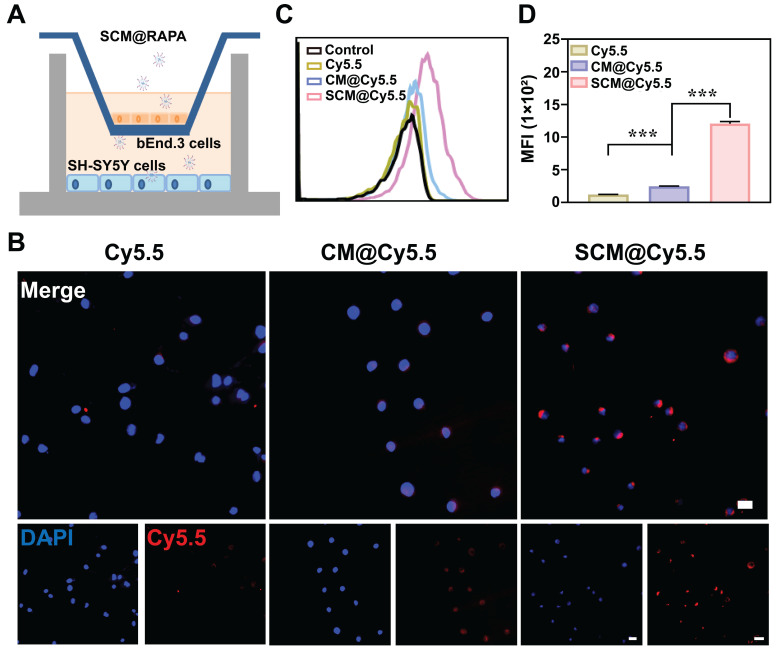
Transportation across the *in vitro* BBB. (A) Schematic representation of *in vitro* BBB model. (B) CLSM images of SH-SY5Y cells in the lower chamber after incubating Cy5.5, CM@Cy5.5, or SCM@Cy5.5 in the upper chamber for 6 h. Scale bar = 20 μm. (C, D) Quantitative analysis of the cellular uptake of SH-SY5Y cells in the lower chamber by FCM (n = 3). Data are presented as mean ± SEM. ***p < 0.001.

Human neuroblastoma cells SH-SY5Y were selected as brain parenchymal cell model to study the cellular internalization of SCM NPs 30. Fluorescent dye Cy5.5 was utilized as a model drug due to their strong fluorescence emission at 690 nm. Figure S3 illustrates the successful internalization of SCM NPs by SH-SY5Y cells, potentially facilitated by the permeability-enhancing capacity of the SS31 peptide 31. The capacity of drug to traverse the BBB is directly linked to the accumulation in the brain, a key determinant of its therapeutic efficacy in central nervous system diseases. To mimic the *in vitro* BBB, a transwell co-culturing system were established. Mouse brain microvascular endothelial cells (bEnd.3) were seeded in the upper chamber, while SH-SY5Y occupied the lower chamber, with both separated by a polycarbonate membrane (Figure 3A). NPs were added to the upper chamber and incubated for 6 h. Subsequent analysis using CLSM and flow cytometry revealed the uptake of NPs by SH-SY5Y cells in the lower chamber. In Figure 3B, minimal red fluorescence was observed in SH-SY5Y cells treated with free Cy5.5, indicating their limited ability to penetrate the BBB independently. However, when treated with SCM@Cy5.5, a significant enhancement in the red fluorescence signal was observed within the SH-SY5Y cells, with the intensity far exceeding that observed in the CM@Cy5.5 treatment cells. Quantitative analysis via flow cytometry further confirmed the enhanced uptake of SCM@Cy5.5 by SH-SY5Y cells (Figure 3C-D). These findings strongly suggest that SS31 peptide modification notably enhances the BBB permeability of SCM@Cy5.5 NPs.

### Impact of SCM@RAPA NPs on autophagic processes in neuronal cells

**Figure 4:**
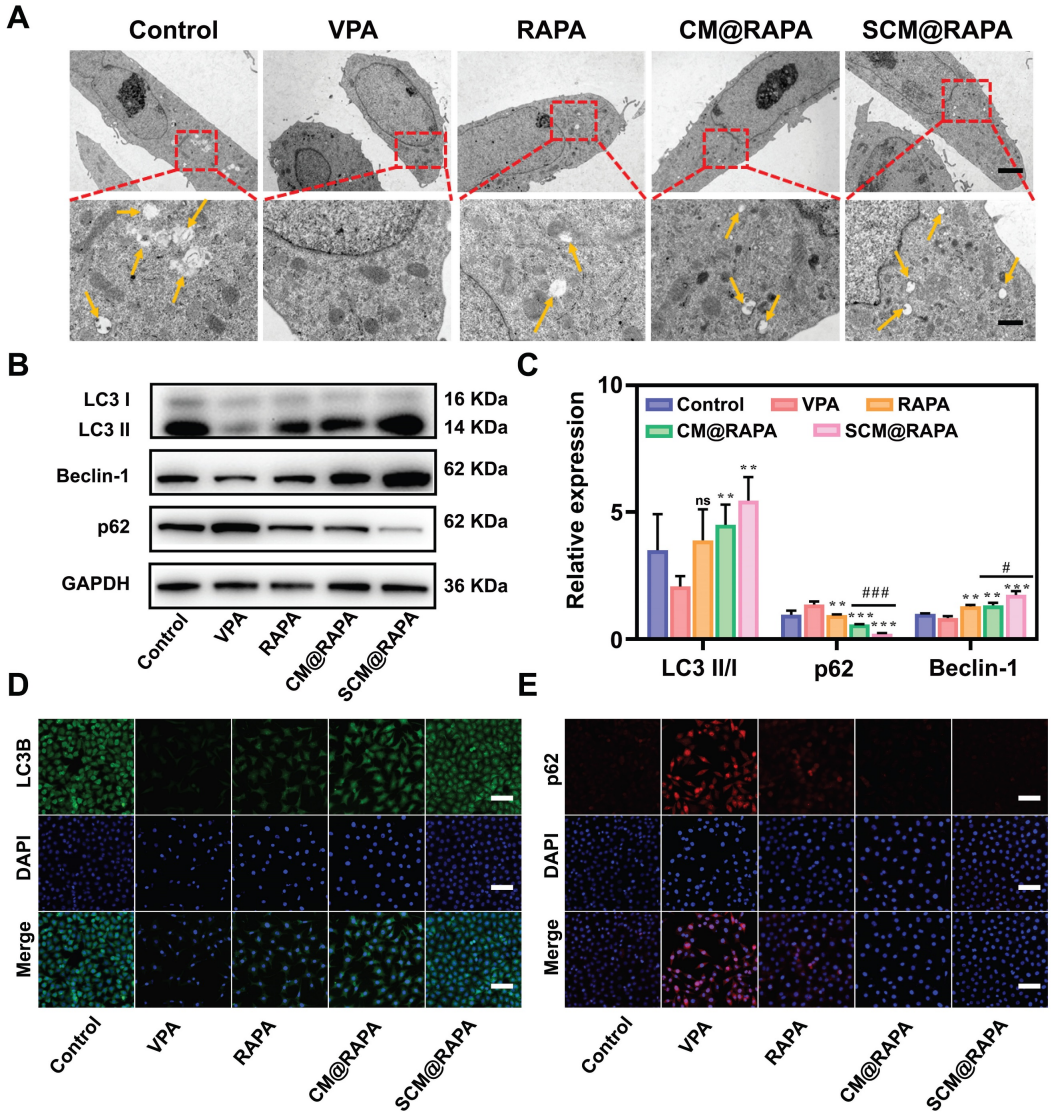
SCM@RAPA increased autophagy levels *in vitro*. (A) TEM images of SH-SY5Y cells with yellow arrows indicating structures of autophagosomes. Scale bar = 5 μm (top) and 1 μm (bottom). (B, C) Western blot images and quantitative analysis of key biomarkers of autophagy after different treatments (n = 3). (D) CLSM of accumulated LC3B (green puncta) in SH-SY5Y cells after different treatments. Scale bar = 80 μm. (E) CLSM of accumulated p62 (red signals) in SH-SY5Y cells after different treatments. Scale bar = 80 μm. Data are presented as mean ± SEM. **p < 0.01 and ***p < 0.001 vs the VPA group. #p < 0.05 and ###p < 0.001 vs CM@RAPA group.

Autophagy plays a crucial role in maintaining neuronal survival and homeostasis, due to neuronal susceptibility to the accumulation of neurotoxic proteins and compromised organelles. Dysregulation of autophagic activity has been associated with perturbations in the PI3K/AKT/mTOR signaling pathway. This aberrant pathway activation is implicated in the pathogenesis of ASD 32,33. Consequently, to validate the influence of SCM@RAPA on autophagic activity in SH-SY5Y cells, the characterization of autophagic structures and the assessment of autophagic marker proteins were conducted. Figures 4A and S5 illustrates the presence of a bilayer membrane structure of autophagosomes in normal cells (indicated by yellow arrow), while autophagosomes disappeared in cells treated with VPA, suggesting a decrease in VPA induced autophagic activity. Conversely, a pronounced increment in autophagosome count was noted in the SCM@RAPA treated cells (Figure S6). Western blot assays corroborated these findings, showing SCM@RAPA markedly increased LC3-II and Beclin-1 expression while reducing p62 levels, indicating enhanced autophagic activity (Figure 4B-C). Immunofluorescence staining further validated these results, revealing decreased green fluorescence corresponding to LC3-II and increased red fluorescence related to p62 in the VPA-induced group. In contrast, the SCM@RAPA-treated group exhibited substantial enhancement in green fluorescence and reduction in red fluorescence (Figure 4D-E, S7, S8). Collectively, these findings substantiated the capacity of SCM@RAPA to efficaciously induce autophagy *in vitro*, highlighting its potential in modulating autophagic processes.

### SCM@RAPA with efficient cytoprotective effect by mitigating oxidative stress

**Figure 5:**
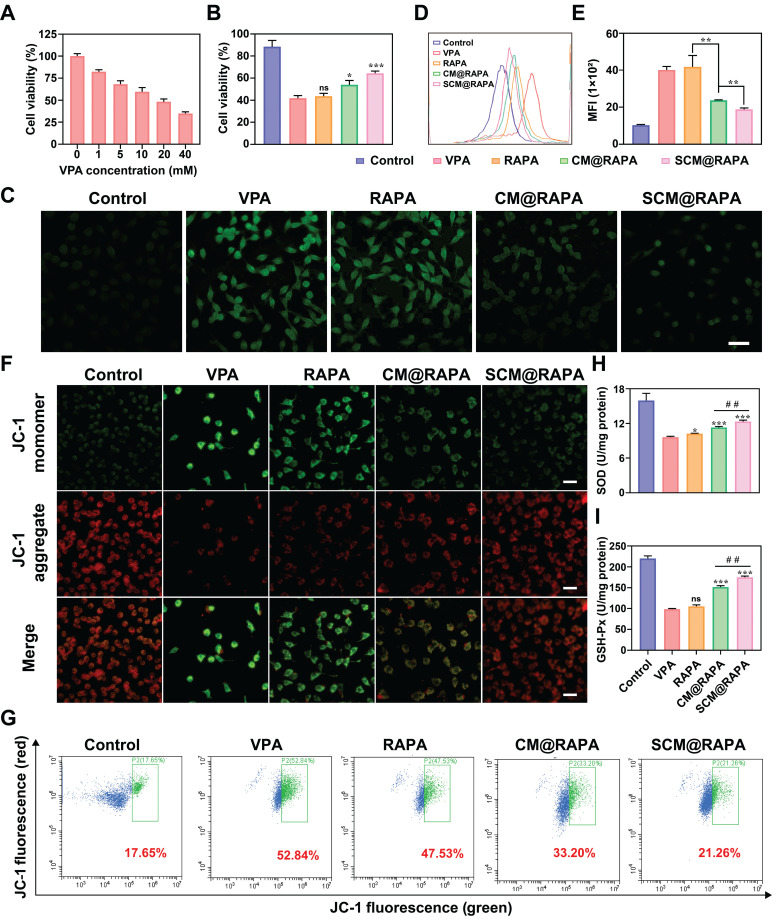
Reduction of VPA-mediated cytotoxicity and oxidative stress by SCM@RAPA NPs. (A) Cell viability of VPA-treated SH-SY5Y cells upon different VPA concentrations (n = 3). (B) Cell viability of VPA-treated SH-SY5Y cells upon different treatments (n = 3). (C) CLSM images of intracellular ROS after different treatments. Scale bar = 50 μm. (D, E) FCM analysis of intracellular ROS after different treatments. (n = 3) (F) CLSM image of JC-1 red-green fluorescence results for different treatment groups. Scale bar = 40 μm. (G) FCM of the intracellular mitochondrial membrane potential, with the ratio of green-fluorescent cells outlined in a box. (H, I) Detection of oxidative damage related biomarkers SOD and GSH-Px after different treatments (n = 3). Data are presented as mean ± SEM. ns indicates no significance, *p < 0.05, **p < 0.01, and ***p < 0.001 vs the VPA group. ##p < 0.01 vs CM@RAPA group.

There is a complex relationship between cellular autophagy and apoptosis. Under stressful conditions, cellular autophagy can shift towards non-autophagic cell death, reducing cell viability 34. VPA treatment inhibits autophagy and induces oxidative stress in autistic rat brains, wherein the mTOR signaling pathway is pivotal 35. To mimic ASD conditions, SH-SY5Y cells were treated with varying VPA concentrations for 24 hours, and cell viability was assessed using the CCK-8 assay. Figure 5A illustrates a VPA concentration-dependent decline in cell viability, reaching 48% at 20 mM VPA, attributable to impaired autophagy induced by VPA. Subsequent treatment of SH-SY5Y cells with 20 mM VPA alongside SCM@RAPA NPs notably augmented cell survival, suggesting the potential of SCM@RAPA in mitigating VPA-induced cytotoxicity (Figure 5B). Moreover, SS31-RBC@RAPA treatment significantly improved VPA-induced morphological changes and adhesion ability decrease in SH-SY5Y cells (Figure S4). Existing studies indicate a correlation between excessive reactive oxygen species (ROS) generation and neuroinflammation triggered by oxidative stress. Such stress levels have been significantly heightened in brain specimens from patients diagnosed with autism 36.

Considering the performance of the SCM@RAPA NPs in reducing VPA cytotoxicity, its potential to scavenge ROS and alleviate oxidative stress was investigated using a DCFH-DA probe. CLSM imaging revealed heightened green fluorescence in VPA-treated SH-SY5Y cells, signifying increased ROS production (Figure 5C). However, intracellular ROS levels showed varying reductions upon exposure to free RAPA, CM@RAPA, or SCM@RAPA, consistent with reports that RAPA can alleviate oxidative stress by activating autophagy 37. Notably, SCM@RAPA-treated cells displayed significantly reduced green fluorescence, indicating substantial ROS scavenging. Flow cytometry analysis supported these findings, suggesting the efficacy of SCM@RAPA NPs in mitigating VPA-induced intracellular ROS levels and subsequent oxidative stress (Figure 5D-E). Mitochondria, crucial for energy synthesis, contribute to about 90% of intracellular ROS production and are susceptible to ROS-induced damage 38. Assessing the mitochondrial membrane potential using the JC-1 mitochondrial membrane potential assay kit revealed VPA-exposed SH-SY5Y cells displayed reduced red fluorescence and increased green fluorescence compared to the control group, indicating VPA-induced mitochondrial damage by diminishing the membrane potential (Figure 5F). While free RAPA showed limited mitigation of mitochondrial dysfunction, SCM@RAPA demonstrated pronounced improvement, suggesting its potential in protecting neurons by reducing ROS levels and preserving normal mitochondrial function, supported by flow cytometry analysis (Figure 5G). SOD and GSH-Px are pivotal in maintaining redox balance. Post SCM@RAPA administration, both SOD and GSH-Px activities were notably restored, highlighting its robust antioxidant effects (Figure 5H-I). Notably, the SS31 peptide, known for its capacity to impede lipid peroxidation, might contribute to these antioxidant effects inherent in SCM@RAPA 21.

### Therapeutic efficacy of SCM@RAPA in ASD rat models

**Figure 6:**
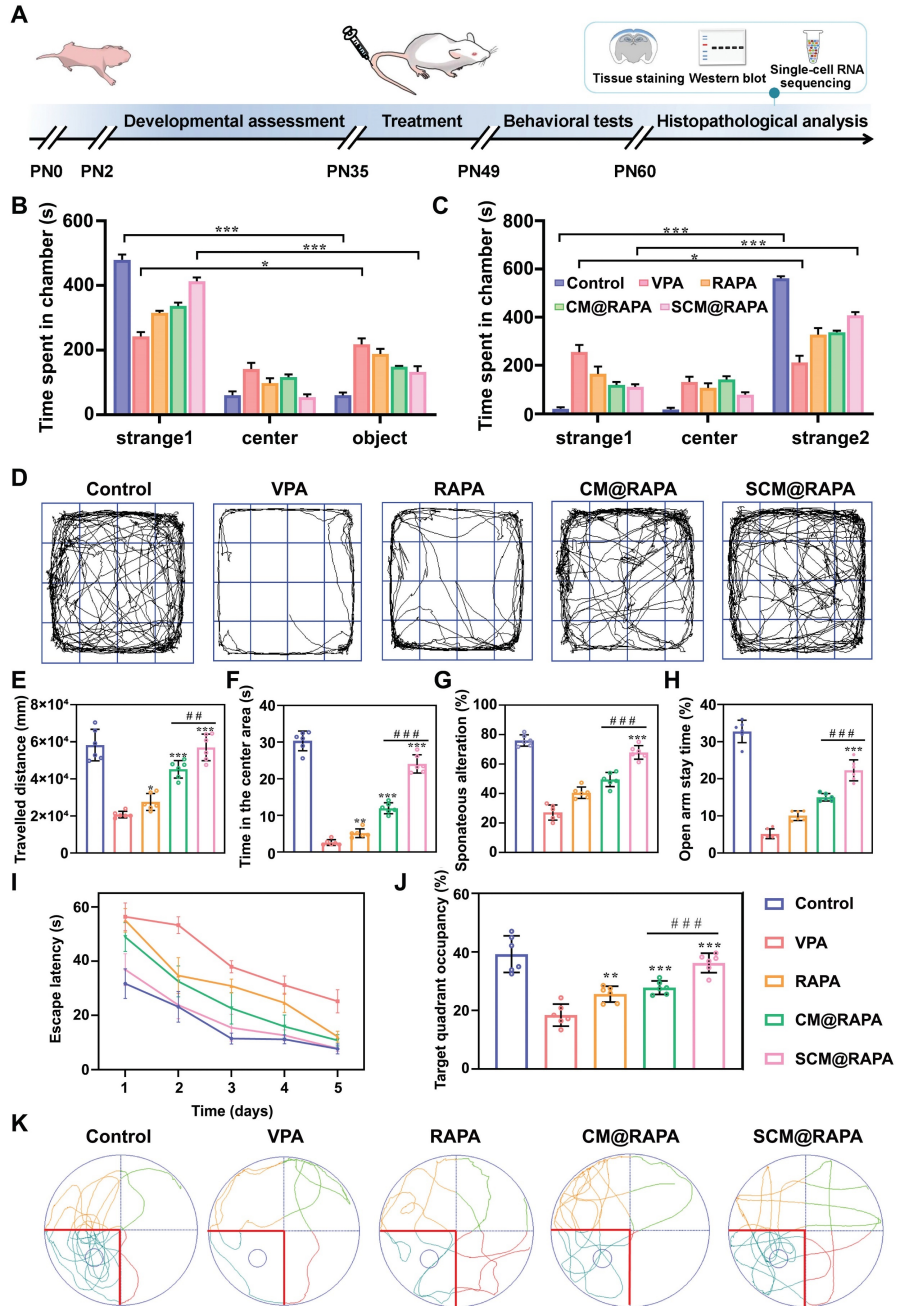
Therapeutic effects of SCM@RAPA *in vivo*. (A) Schematic description of the experimental timeline for the prenatal VPA-induced ASD rat model, and treatment by SCM@RAPA with behavioral tests and biological assessments. (B,C) Quantitation of time spend in each chamber in the first stage and second stage (n = 6). (D) Trajectories and quantitation of (E) total travelled distance and (F) time in the center area in open field test (n = 6). (G) Quantitation of spontaneous alternation rate in Y maze (n = 6). (H) Quantitation of percentage of time spent in the elevated plus maze open arm (n = 6). (I) Quantitation of the escape latencies, (J) time spent in the target quadrant and (K) trajectories in Morris water maze (MWM) test (n = 6). Data are presented as mean ± SEM. *p < 0.05, **p < 0.01, and ***p < 0.001 vs the VPA group. ##p < 0.01 and ###p < 0.001 vs CM@RAPA group.

To further probe the therapeutic impact of SCM@RAPA on ASD *in vivo*, an ASD rat model was initially established via a single intraperitoneal administration of VPA on the 12.5^th^ day of gestation. Negative geotaxis, plane correction, cliff avoidance and swimming score tests were performed to evaluate the physiological development of the offspring of male rats to determine the successful establishment of the animal model (Figure S9). Subsequently, RAPA, CM@RAPA and SCM@RAPA were intravenously administered daily to 35-day-old ASD rats for 14 days. Post-treatment, behavioral experiments were performed to assess the protective effect of SCM@RAPA against social dysfunction and memory deficits mediated by VPA administration (Figure 6A). The three-chamber test evaluated social traits, revealing SCM@RAPA's significant improvement in time spent with unfamiliar rats (strangers 1 and 2), indicating enhanced social ability and novelty (Figures 6B-C). Subsequently, open field tests were carried out to assess movement and anxiety behavior of ASD rats. SCM@RAPA NPs exhibited the most efficacious alleviation of anxiety symptoms in the VPA-induced rat model. This was evidenced by enhanced activity within the central region post-treatment, a behavior akin to that observed in the control group (Figure 6D-F). Furthermore, the Y-maze and self-grooming were utilized to evaluate repetitive behavior. SCM@RAPA treatment reversed the VPA-induced declines in spontaneous alternation rate, while decrease the number and duration of VPA-induced self-grooming, suggesting its potential in alleviating ASD-related repetitive behaviors (Figure 6G, S10). The elevated plus maze test indicated the improvement of open arm duration by SCM@RAPA treatment, suggesting its potential in alleviating ASD-related anxiety (Figure 6H). Finally, the MWM test was employed to investigate the capacity of SCM@RAPA NPs to rectify cognitive deficits observed in ASD rat models. Over the course of the 5-day directional navigation experiment, a marginal improvement in cognitive performance was observed in the free RAPA and CM@RAPA treatment groups compared to the VPA group. However, the SCM@RAPA-treated rats exhibited a latency akin to the control group, suggesting their superior efficacy in ameliorating cognitive deficits in ASD rats (Figure 6I). Upon removal of the platform, rats treated with SCM@RAPA spent the longest time in the target quadrant, exhibiting superior memory retention compared to other groups (Figure 6J-K).

### Brain penetration and neuroprotective effects of SCM@RAPA

**Figure 7:**
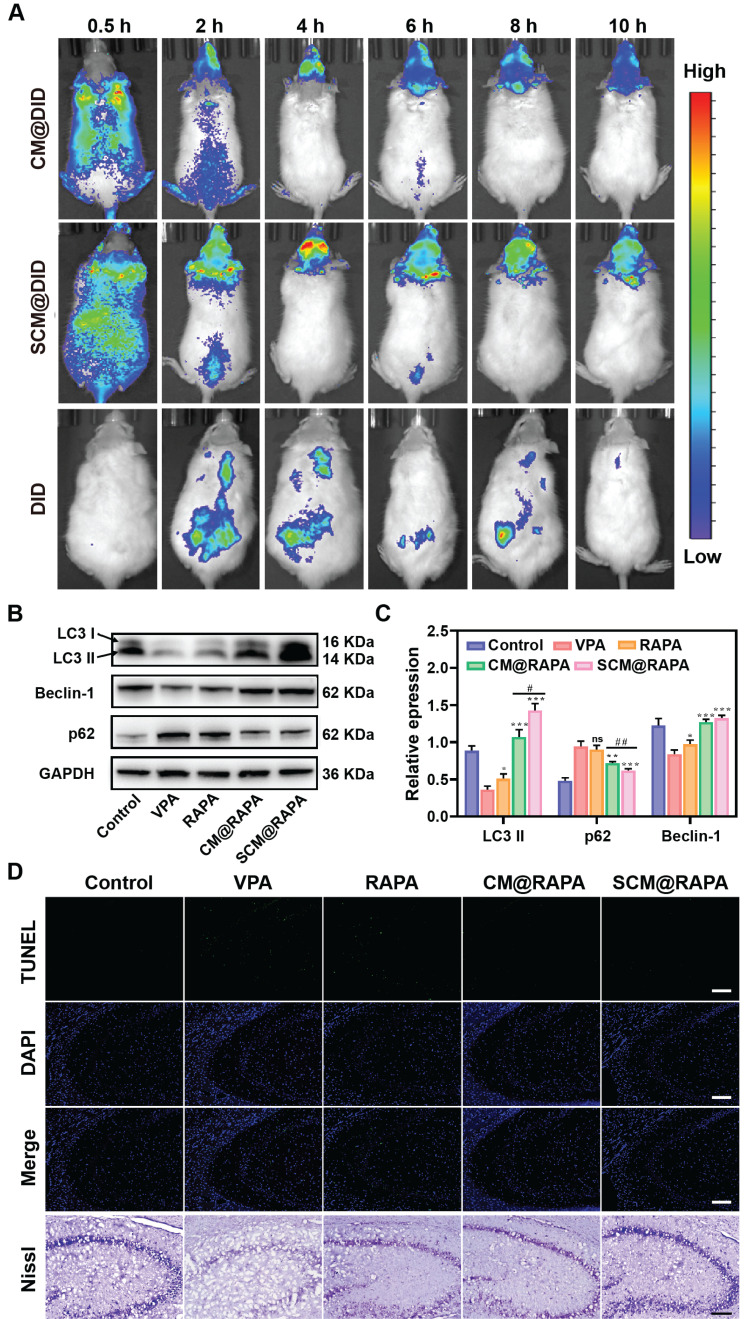
SCM@RAPA across the BBB and exert neuroprotective effects by activating autophagy. (A) *In vivo* NIR fluorescence imaging of VPA rats at different time points after tail vein injection of CM@DID, SCM@DID or DID. (B, C) Western blot images and quantitative analysis of key biomarkers of autophagy in the hippocampus of ASD rats after different treatments (n = 3). (D) Images of TUNEL staining and Nissl staining of hippocampal sections. Scale bar = 100 μm (TUNEL) and 200 μm (Nissl). Data are presented as mean ± SEM. *p < 0.05, **p < 0.01, and ***p < 0.001 vs the VPA group. #p < 0.05 and ##p < 0.01 vs CM@RAPA group.

The therapeutic efficacy of SCM@RAPA NPs is intricately linked to their capacity to efficiently traverse the BBB. Real-time fluorescence imaging analysis was conducted to assess the brain-targeting capability of these NPs *in vivo*. Figure 7A depicts a noticeable fluorescence signal observed in the brain 2 h post-injection in the SCM@DiD group, peaking at around 4 h post-injection. Notably, the fluorescence intensity in the brain of the CM@DiD group was consistently lower than that of the SCM@DiD group at all measured time points, indicating the significant enhancement of NPs permeability across the BBB facilitated by the SS31 peptide. Studies have highlighted compromised autophagy functionality in the brains of individuals with autism, with the activation of autophagy effectively ameliorating typical autism symptoms 39. Consequently, the effect of SCM@RAPA on autophagy activation *in vivo* was investigated. As expected, VPA treatment resulted in a downregulation of autophagy levels. However, the SCM@RAPA treatment group effectively reversed this VPA-induced decline in autophagy *in vivo* compared to other groups (Figure 7B-C). TUNEL staining revealed significant neuronal apoptosis in the hippocampus of VPA-treated rats, which was notably reversed with SCM@RAPA treatment (Figure 7D). Nissl staining analysis indicated a scarcity of Nissl bodies and conspicuous shrinkage and fragmentation in the neuronal morphology of VPA-treated rats compared to control rats. However, SCM@RAPA treatment significantly ameliorated neuronal loss and shrinkage. Additionally, histological analysis via H&E staining demonstrated preserved tissue integrity in the treatment group, devoid of discernible tissue damage, affirming the favorable biosafety profile of SCM@RAPA (Figure S11).

### Single-cell RNA sequencing

**Figure 8:**
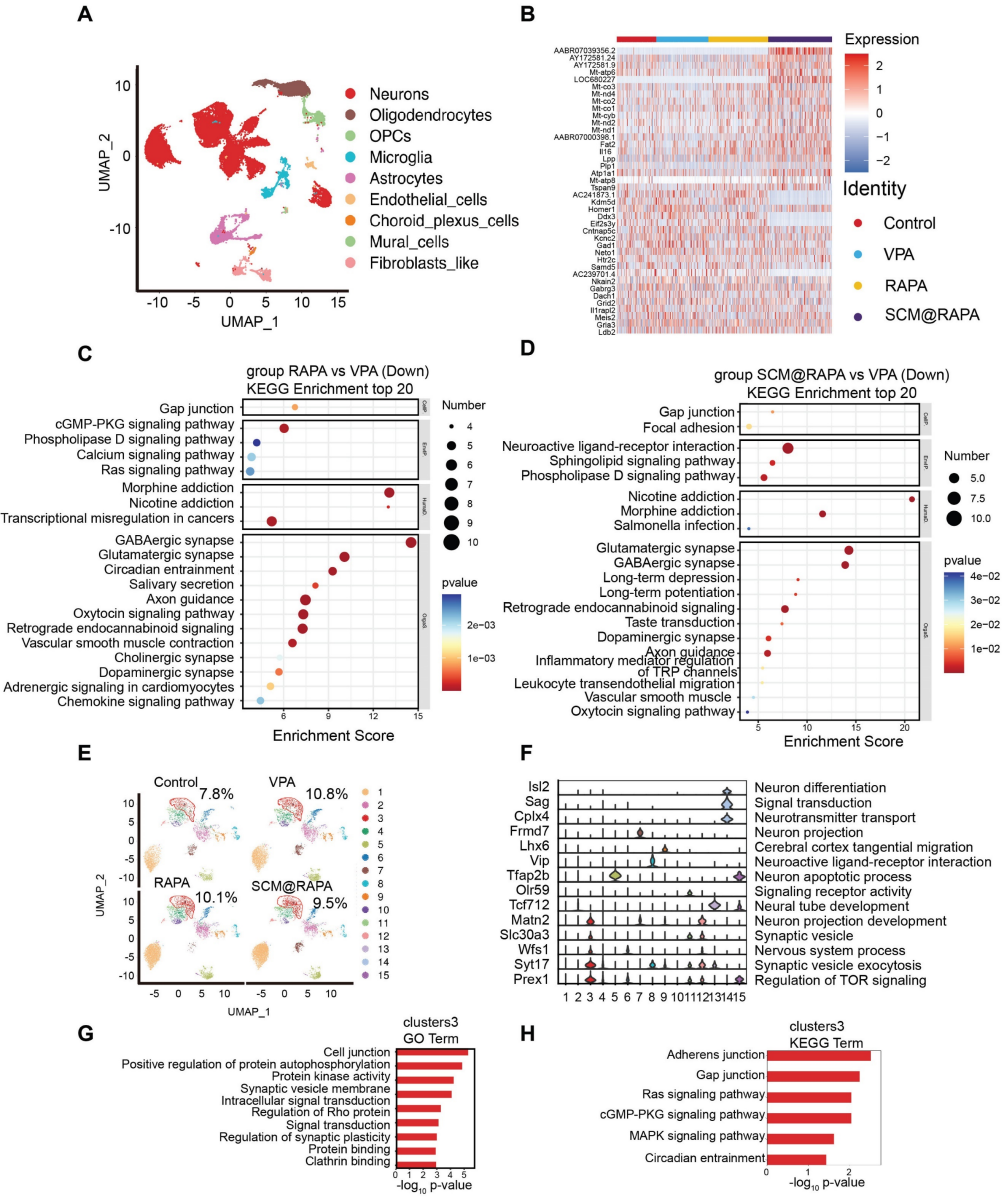
Single-cell RNA sequencing. (A) Uniform manifold approximation and projection (UMAP) plot of different cell types (B) Heatmap of top 20 DEGs in VPA group and SCM@RAPA group. (C) Bubble Chart of KEGG pathways that downregulated in the RAPA group vs VPA group. (D) Bubble Chart of KEGG pathways that downregulated in the SCM@RAPA group vs VPA group. (E) UMAP plot of subclusters of neurons. (F) Violin plots of top marker genes in specific neurons subclusters. (G) Bar chart of GO terms enriched in subcluster 3 of neurons. (H) Bar chart of KEGG terms enriched in subcluster 3 of neurons.

Brain tissue samples treated with SCM@RAPA were subjected to Single-cell RNA sequencing (scRNA-seq) to elucidate specific biological changes and signaling pathways. Following quality-control filtering, scRNA profiles were obtained from 9,981 to 13,792 cells, classified into distinct cell types, including neurons, oligodendrocytes, OPCs, microglia, astrocytes, endothelial cells, choroid plexus cells, mural cells, and Fibroblast-like cells (Figure 8A).

Notably abundant in neurons, differential gene expression analysis focused on this cell type across groups (Figure S12, S13). The VPA group exhibited 115 DEGs, with 33 up-regulated and 82 down-regulated genes compared to the Control group. Conversely, the RAPA group displayed 138 up-regulated and 171 down-regulated genes relative to the VPA group. Meanwhile, the SCM@RAPA group presented 154 up-regulated and 102 down-regulated genes (Table S1). Heatmap plotting of the top 20 genes by fold change highlighted distinctions in genes linked to the mTOR signaling pathway (*AC239701.4*), cAMP signaling pathway (*Atp1a1, Gria3*), and GABAergic synapse (*Gabrg3, Gad1, Homer1*) across these groups (Figure 8B). SCM@RAPA NPs treatment upregulated mitochondrial gene expression (*Mt-co3, Mt-nd4, Mt-co2, Mt-co1, Mt-cyb, Mt-nd2, Mt-nd1, Mt-atp8*) compared to the VPA and RAPA groups, suggesting improved mitochondrial function. The results of Gene Ontology (GO) enrichment and Kyoto Encyclopedia of Genes and Genomes (KEGG) analysis suggested that, compared to the Control group, the VPA group exhibited alterations in synaptic function, downregulation of autophagy-related genes, and upregulation of genes associated with dopaminergic synapse and glutamatergic synapse (Figure S14-16). These findings indicate abnormal synaptic function and reduced autophagy in the VPA group. KEGG enrichment analysis further revealed that, compared to the VPA group, treatment with RAPA and SCM@RAPA mitigated the overactivation of the nervous system, particularly in the activity of glutamatergic and GABAergic synapses (Figure 8C-D). Additionally, the SCM@RAPA group demonstrated functional downregulation in dopamine synapses, long-term depression, and long-term potentials. Gene Set Variation Analysis (GSVA) revealed that among the top 10 enriched pathways, SCM@RAPA negatively regulated neural impulse transmission (Figure S17). These results highlight the inhibitory effects of SCM@RAPA on synaptic overactivation and its potential intervention in depressive emotions.

The secondary analysis of neuronal subtypes resulted in 15 subtypes with different functions. Major functions of each subcluster were found by analyzing specific top gene markers, including neuronal function (*Isl2, Frmd7, Tfap2b*), synaptic function (*Slc30a3*, *Syt17*), signal transduction (*Sag, Olr59*) and nervous system process (*Wfs1*). In all subtypes, the proportion of cluster 3 in the VPA group exceeded that in the other groups, gradually decreasing in the RAPA and SCM@RAPA groups (Figure 8E). Marker gene (*Prex1*) of cluster 3 regulates TOR signaling, aligning with our aforementioned results (Figure 8F). Therefore, RAPA exerts its effects by inhibiting the TOR signaling pathway, resulting in functional alterations in neurons and synapses.

Furthermore, the GO and KEGG enrichment analysis on the top 200 markers of cluster 3 revealed their functions concentrated in signal transduction and cellular community (Figure 8G-H). Interestingly, cluster 3 is associated with the Ras signaling pathway and MAPK signaling pathway, which was associated with cell apoptosis 40. Autophagy raises the stress threshold required to induce cell death through multiple mechanisms and selectively eliminates pro-apoptotic signal transducers, thereby blocking apoptosis 41. Our research findings demonstrate that SCM@RAPA enhances autophagy by suppressing mTOR activation, consequently suppressing apoptosis. This may explain the reduction in the number of cell clusters in cluster 3 following treatment. Further investigation will delve into the impact of SCM@RAPA on cell apoptosis in cluster 3 and its effects on synaptic energy.

## Conclusions

In summary, we proposed and engineered a red blood cell membrane-mimetic RAPA nano-delivery system with the ability to traverse the BBB for autism treatment. SCM@RAPA nanoparticles exhibit excellent oxidative stress-responsive release characteristics and a significantly enhanced ability to penetrate the BBB, leading to substantial drug accumulation in brain tissues. Furthermore, SCM@RAPA nanoparticles effectively protect neurons from VPA-induced damage by activating autophagic pathways, alleviating oxidative stress, and preserving mitochondrial function, as corroborated by single-cell RNA sequencing results. Notably, behavioral experiments in ASD rat models demonstrate significant improvements in social dysfunction, anxiety, repetitive behaviors, cognitive deficits, and memory retention. The outcomes of this study establish a novel theoretical foundation and offer a fresh perspective on utilizing biomimetic SCM@RAPA nanoparticles with autophagy-activating and oxidative stress-regulating abilities in the treatment of ASD.

## Supplementary Material

Supplementary figures and table.

## Abbreviations

ASD: autism spectrum disorder
BBB: blood-brain barrier
BCA: bicinchoninic acid
CCK-8: cell counting kit-8 assay
CLSM: confocal laser scanning microscopy
CM: cell membrane
DCFH-DA: 2,7-dichlorodihydrofluorescein diacetate
DEGs: differentially expressed genes
DiD: 1,1'-dioctadecyl-3,3,3',3'-tetramethylindodicarbocyanine,4-chlorobenzenesulfonate salt
DLS: dynamic light scattering
FT-IR: fourier transform infrared
GAPDH: glyceraldehyde-3-phosphate dehydrogenase
GO: gene ontology
GSH-Px: glutathione peroxidase
GSVA: gene set variation analysis
HR: hemolysis rate
HRP: horseradish peroxidase
KEGG: Kyoto encyclopedia of genes and genomes
MMP: mitochondrial membrane potential
MWM: Morris water maze
NPs: nanoparticles
PCA: principal component analysis
PI3K: phosphoinositide-3-kinase
PMSF: phenyl methane sulfonyl fluoride
PN: postnatal
PVDF: polyvinylidene difluoride
RAPA: rapamycin
RIPA: radioimmunoprecipitation assay
ROS: reactive oxygen species
SCM: DSPE-PEG-SS31-CM
scRNA-seq: single-cell RNA sequencing
SD: standard deviation
SDS-PAGE: sodium dodecyl sulphate-polyacrylamide gel electrophoresis
SOD: standard superoxide dismutase
TdT: terminal deoxynucleotidyl transferase
TEM: transmission electron microscope
UMAP: uniform manifold approximation and projection
UMI: unique molecular identifier
VPA: valproic acid
XPS: X-ray photoelectron spectroscopy
