# Supplementary material for: Biomimetic nanoparticles with enhanced rapamycin delivery for autism spectrum disorder treatment via autophagy activation and oxidative stress modulation
Source: Theranostics. 2024 Jul 15;14(11):4375–92. doi: 10.7150/thno.95614 (PMC11303075; doi:10.7150/thno.95614)
Supplement: Supplementary file 1 — Supplementary figures and table. [file thnov14p4375s1.pdf]

Supplementary data

Biomimetic nanoparticles with enhanced rapamycin delivery for autism spectrum disorder treatment via autophagy activation and oxidative stress modulation

Chenlin Miao[1,§], Yizhe Shen[1,§], Yue Lang[1], Hui Li[1,2], Yan Gong[1], Yamei Liu[1], Huafei Li[1], Byron C. Jones[3], Fuxue Chen[1,*], and Shini Feng[1,*]

[1] School of Lifesciences, Shanghai University, 333 Nanchen Road, 200444, Shanghai, P.R.C.

[2] School of Environmental and Chemical Engineering, Shanghai University, 333 Nanchen Road, 200444, Shanghai, P.R.C.

[3] Department of Genetics, Genomics and Informatics, University of Tennessee Health Science Center, Memphis

[*]Email: chenfuxue@staff.shu.edu.cn; fengshini@shu.edu.cn

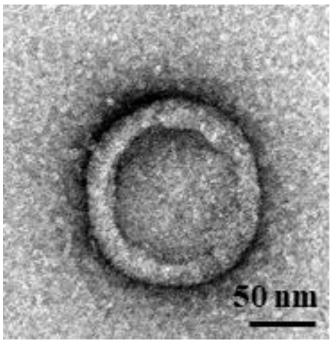

Figure S1. TEM images of SCM@RAPA. Scale bar = 50 nm.

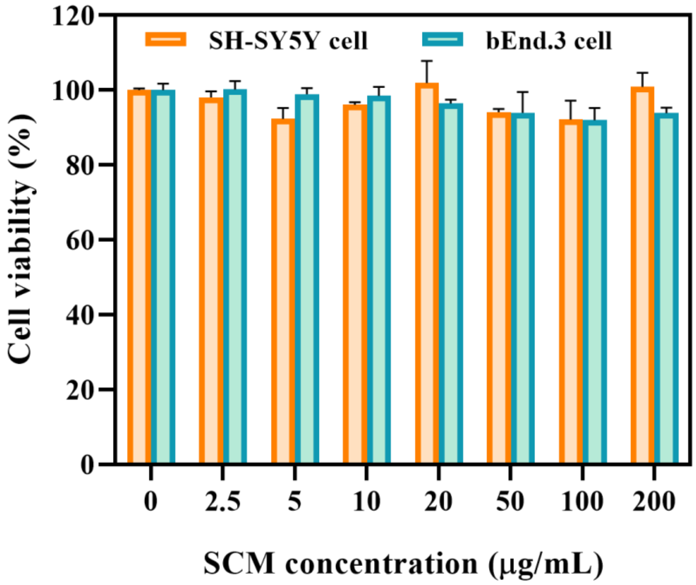

Figure S2. Cytotoxicity of SCM to SH-SY5Y and bEnd.3 cells with different SCM concentration for 24 h (n = 3). Data are presented as mean ± SEM.

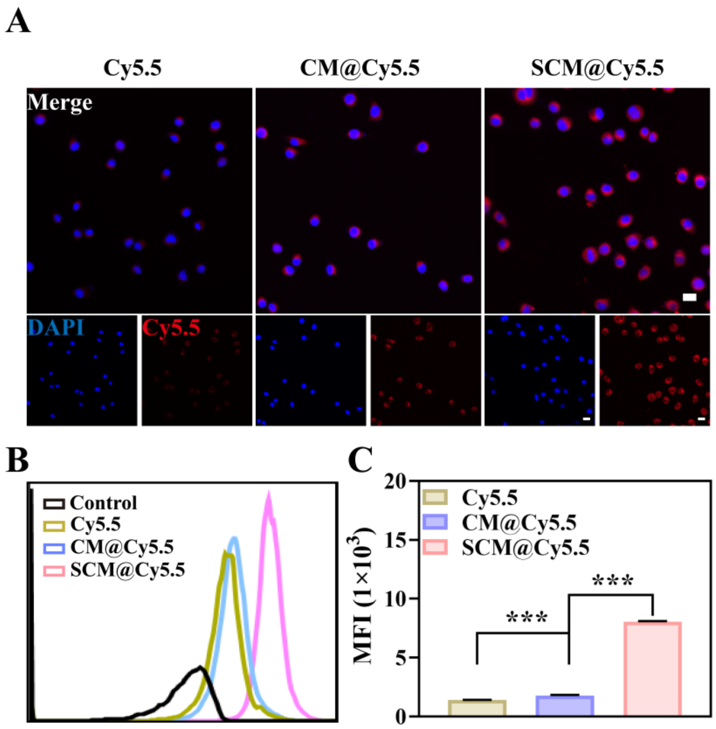

Figure S3. Cellular uptake of NPs. (A) CLSM images of SH-SY5Y cells incubated with Cy5.5, CM@Cy5.5 and SCM@Cy5.5 for the same time periods. Scale bar = 20 μm. (B, C) Quantitative analysis of SH-SY5Y cell uptake by FCM for the same time periods (n = 3). Data are presented as mean ± SEM. ***$p < 0.0001$.

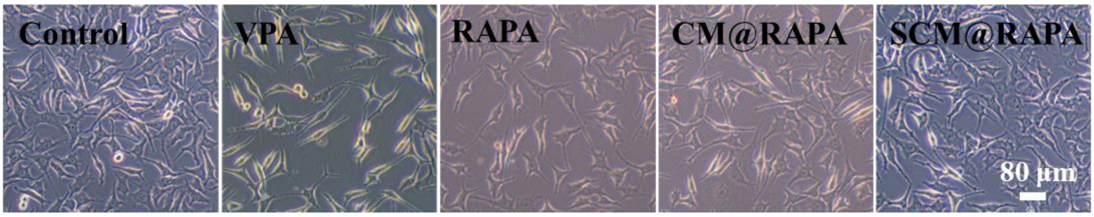

Figure S4. Images of changes in cell morphology under VPA and different NPs (RAPA, CM@RAPA, SCM@RAPA) treatments for 24 h. Scale bar = 80 μm.

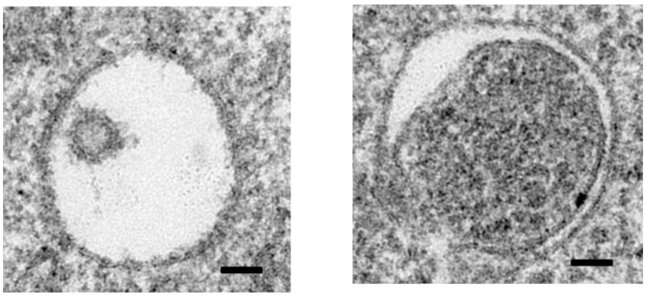

Figure S5. TEM images of SH-SY5Y cells in the control group with bilayer membrane structures of autophagosomes. Scale bar = 50 nm.

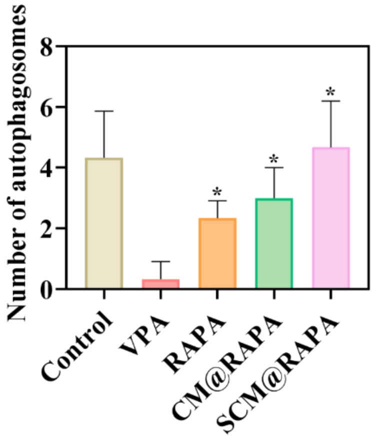

Figure S6. Statistical analysis of autophagosome quantity (n = 3). Data are presented as mean ± SEM. *$p < 0.05$ vs the VPA group.

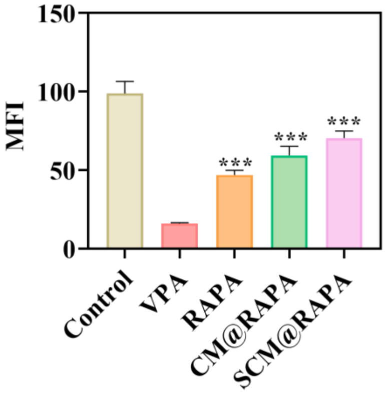

Figure S7. Statistical chart of average fluorescence intensity of LC3B (n = 3). Data are presented as mean ± SEM. ***p < 0.001 vs the VPA group.

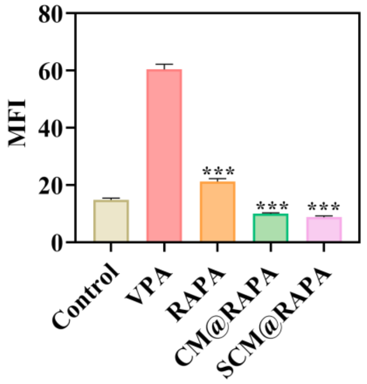

Figure S8. Statistical chart of average fluorescence intensity of p62 (n = 3). Data are presented as mean ± SEM. ***$p < 0.001$ vs the VPA group.

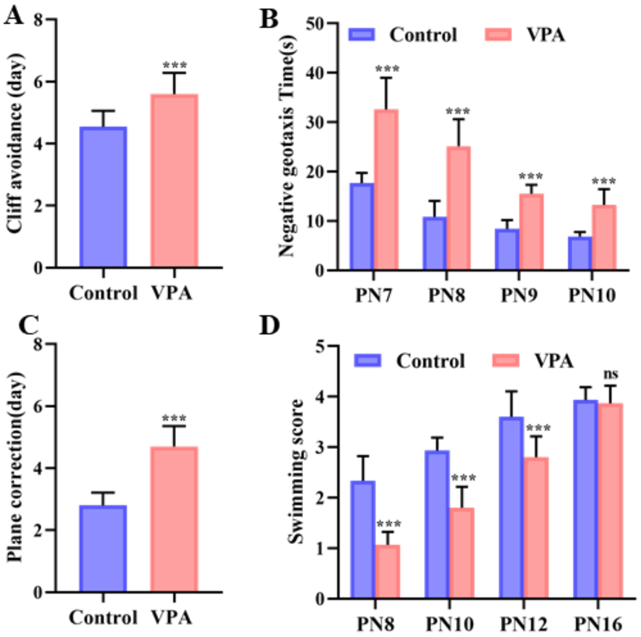

Figure S9. Detection of physiological and neurobehavioral development indicators in ASD rats. Quantification of (A) cliff avoidance, (B) negative geotaxis, (C)plane correction, and (D) swimming score (n = 10). Data are presented as mean ± SEM. ns indicates no significance, ***p < 0.001.

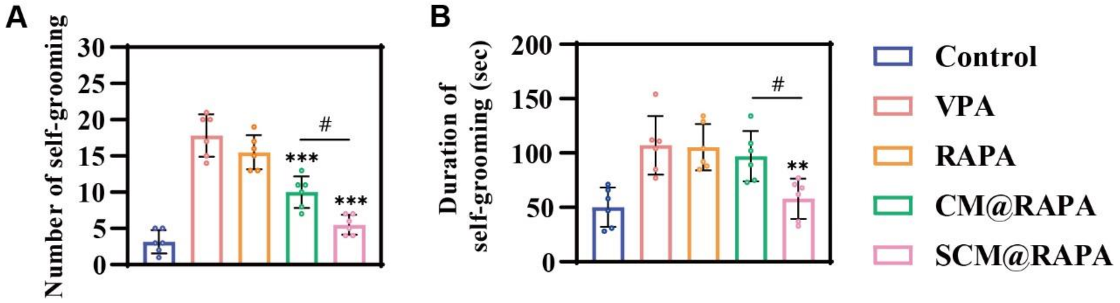

Figure S10. Quantitation of (A) number and (B) duration of self-grooming (n = 6). Data are presented as mean ± SEM. $^{**}p < 0.01$ and $^{***}p < 0.001$ vs the VPA group. $^{\#}p < 0.05$ vs CM@RAPA group.

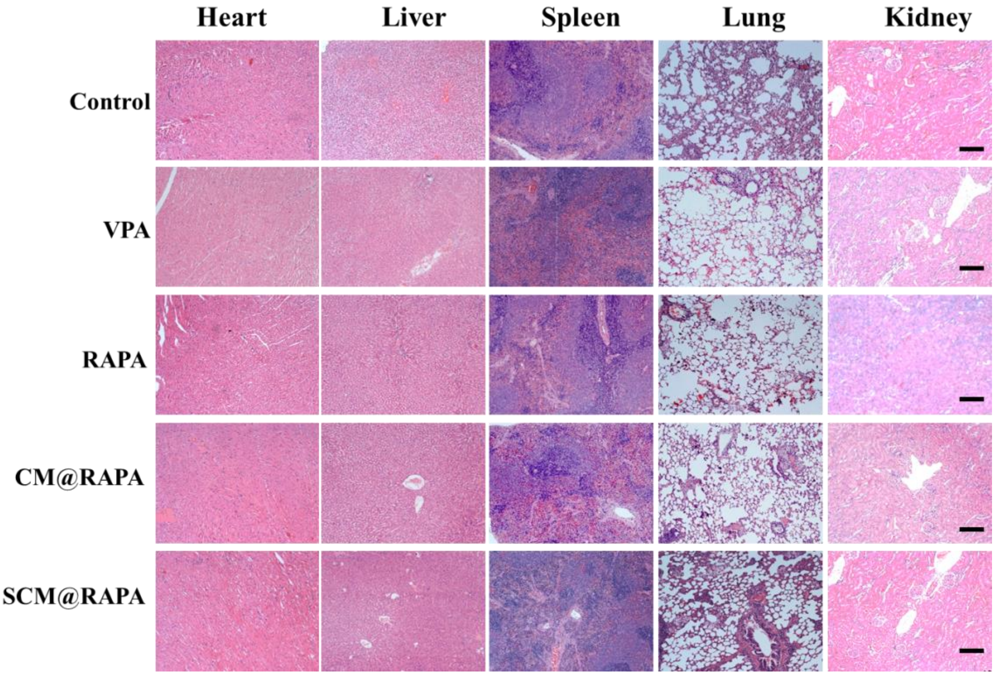

Figure S11. In vivo biocompatibility analysis. H&E staining of heart, liver, spleen, lung, and kidney tissue sections from rats in the indicated treatment groups. Scale bar = 100 μm.

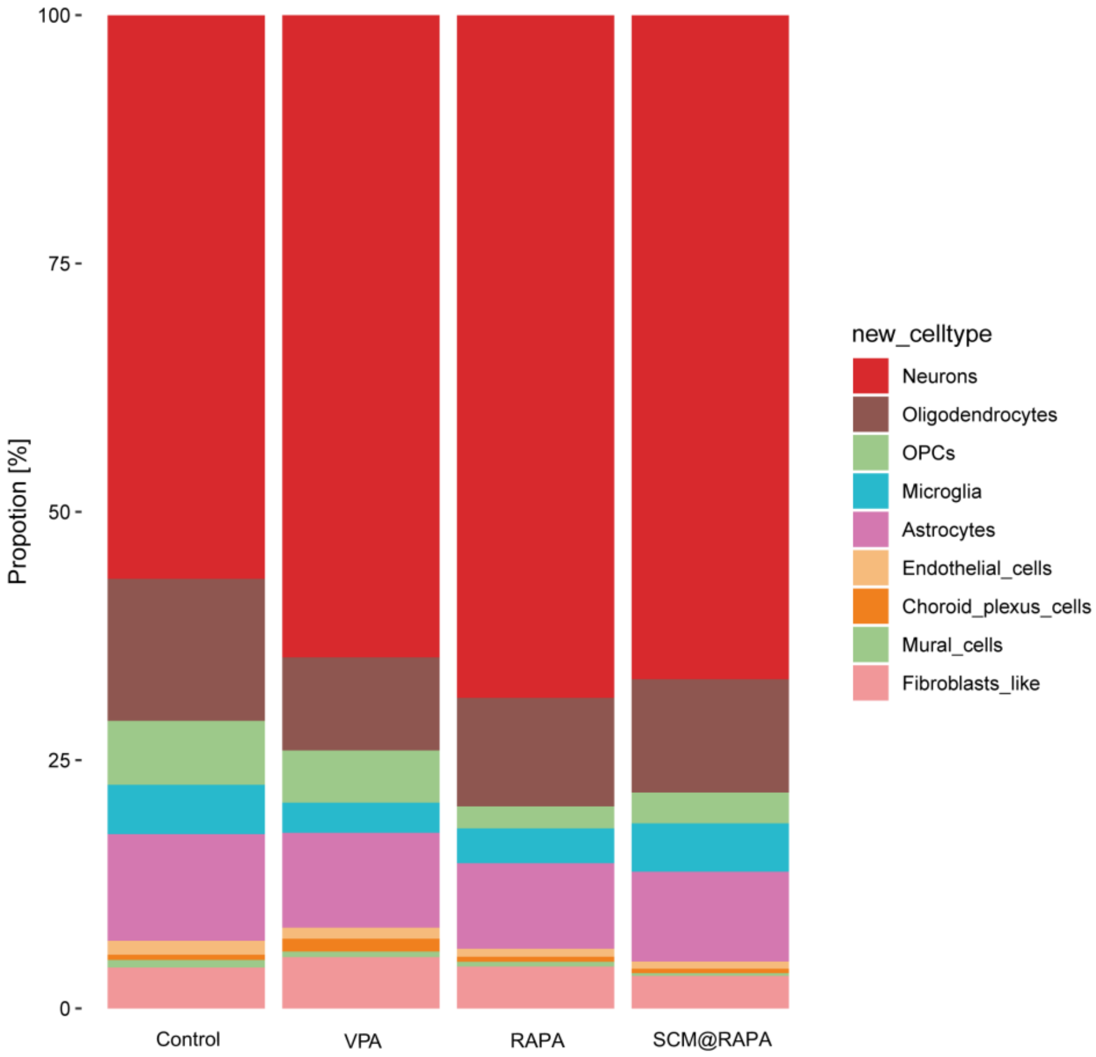

Figure S12. Statistical chart of the proportion of different cell types in Control, VPA, RAPA and SCM@RAPA group.

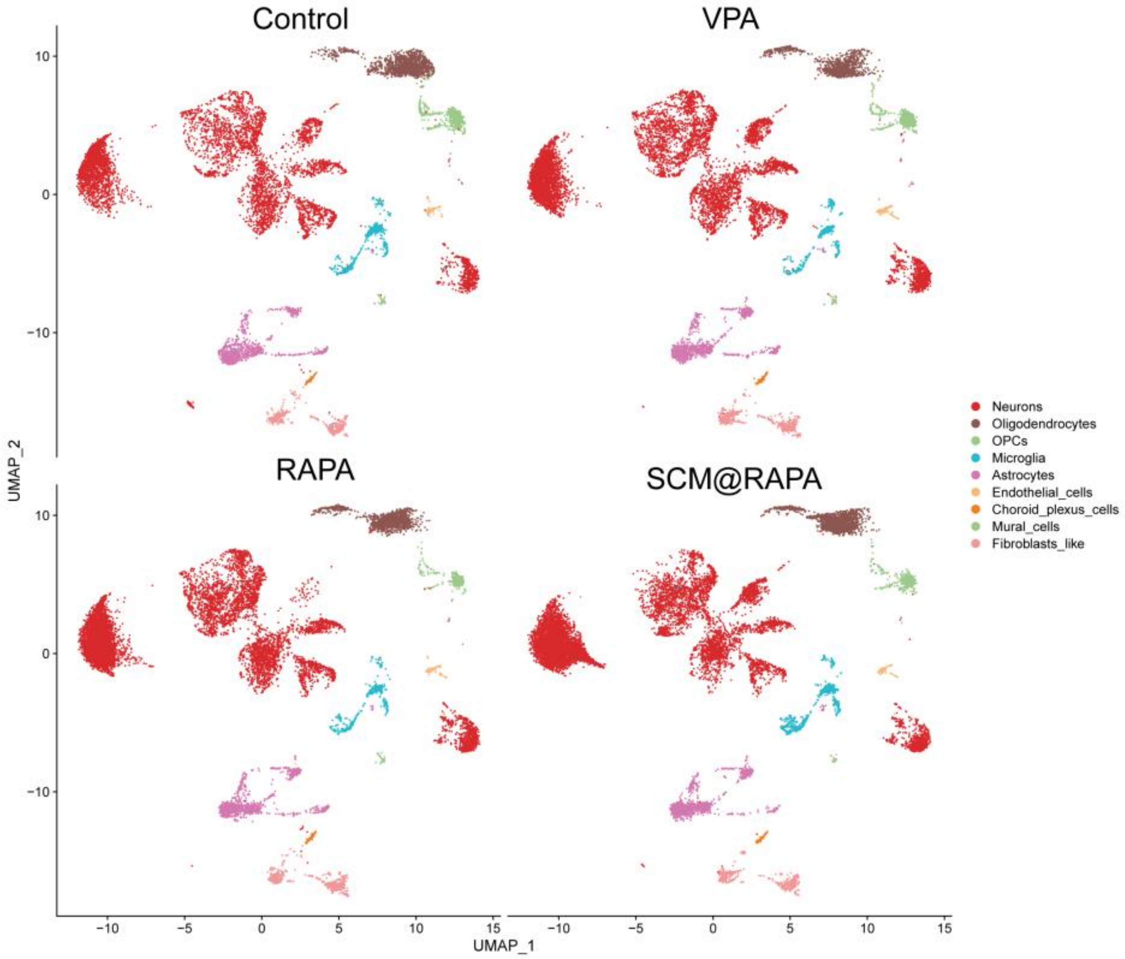

Figure S13. UMAP plot of different cell types in Control, VPA, RAPA and SCM@RAPA group.

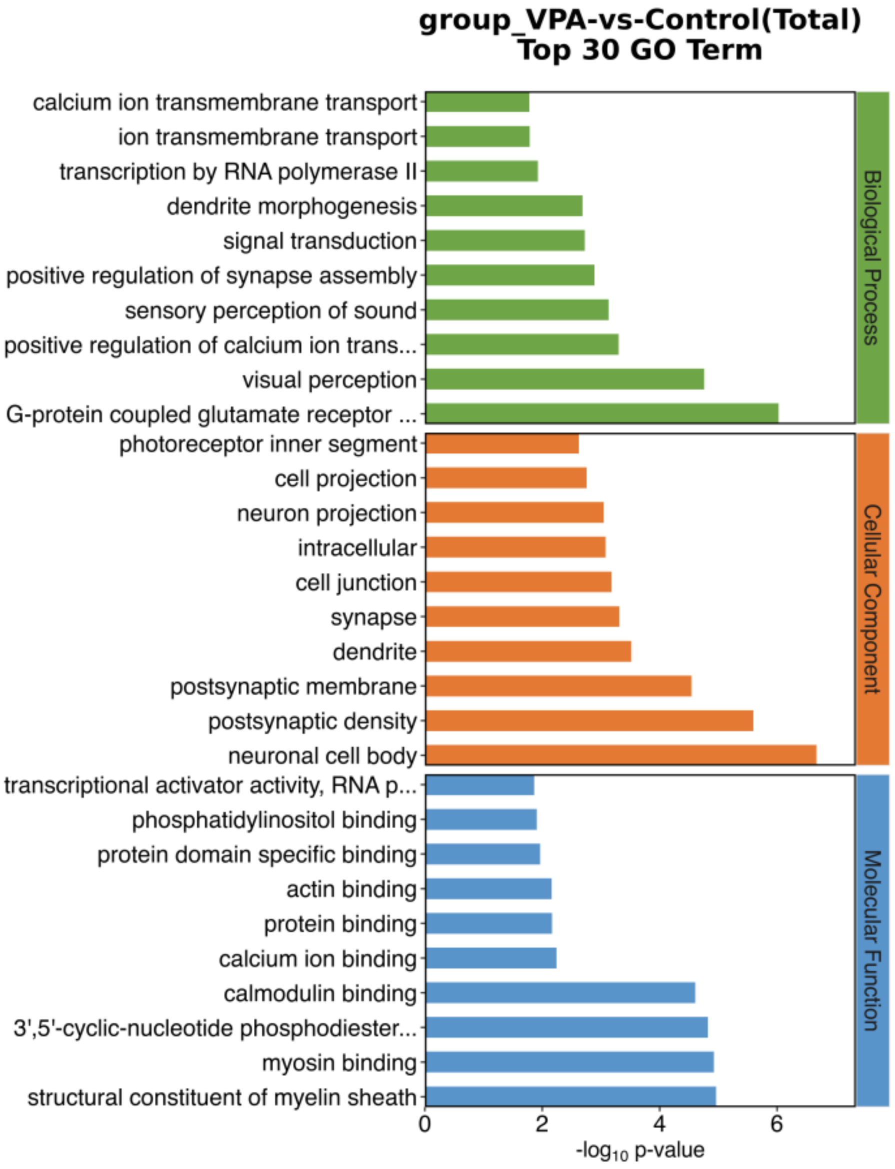

Figure S14. Bar chart of GO terms enriched in the VPA group vs Control group.

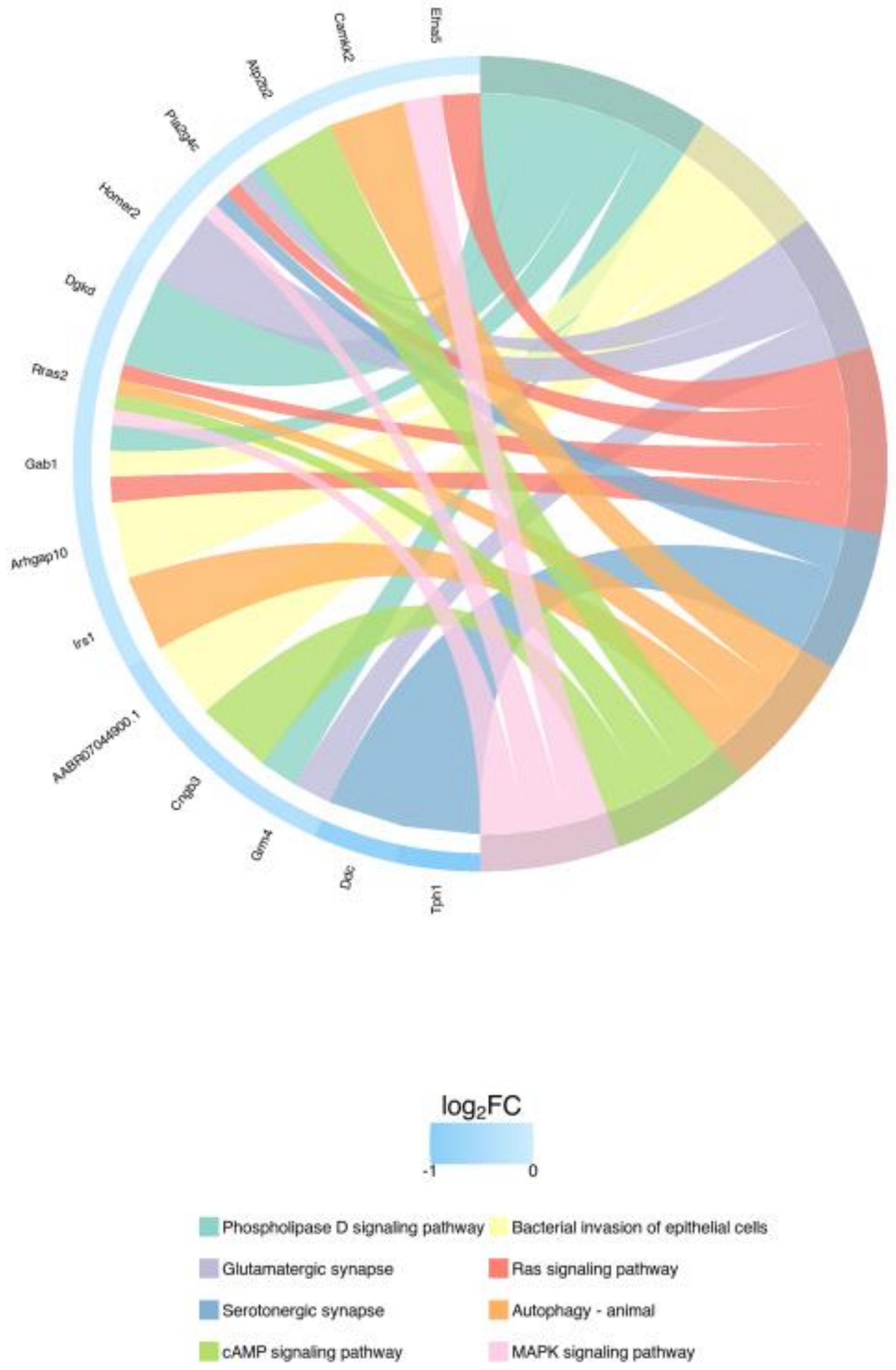

Figure S15. Chord diagram of pathways that downregulated in the VPA group vs Control group.

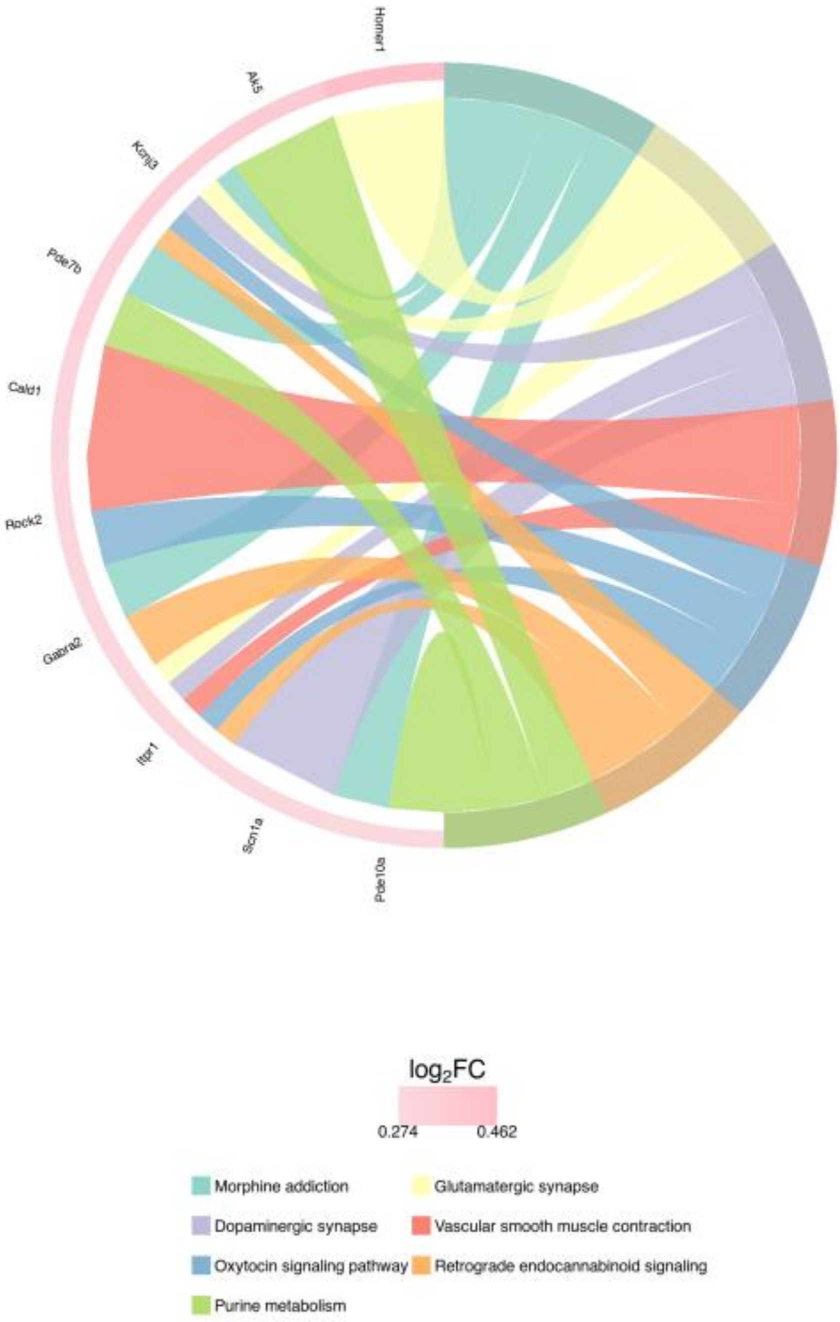

Figure S16. Chord diagram of pathways that upregulated in the VPA group vs Control group.

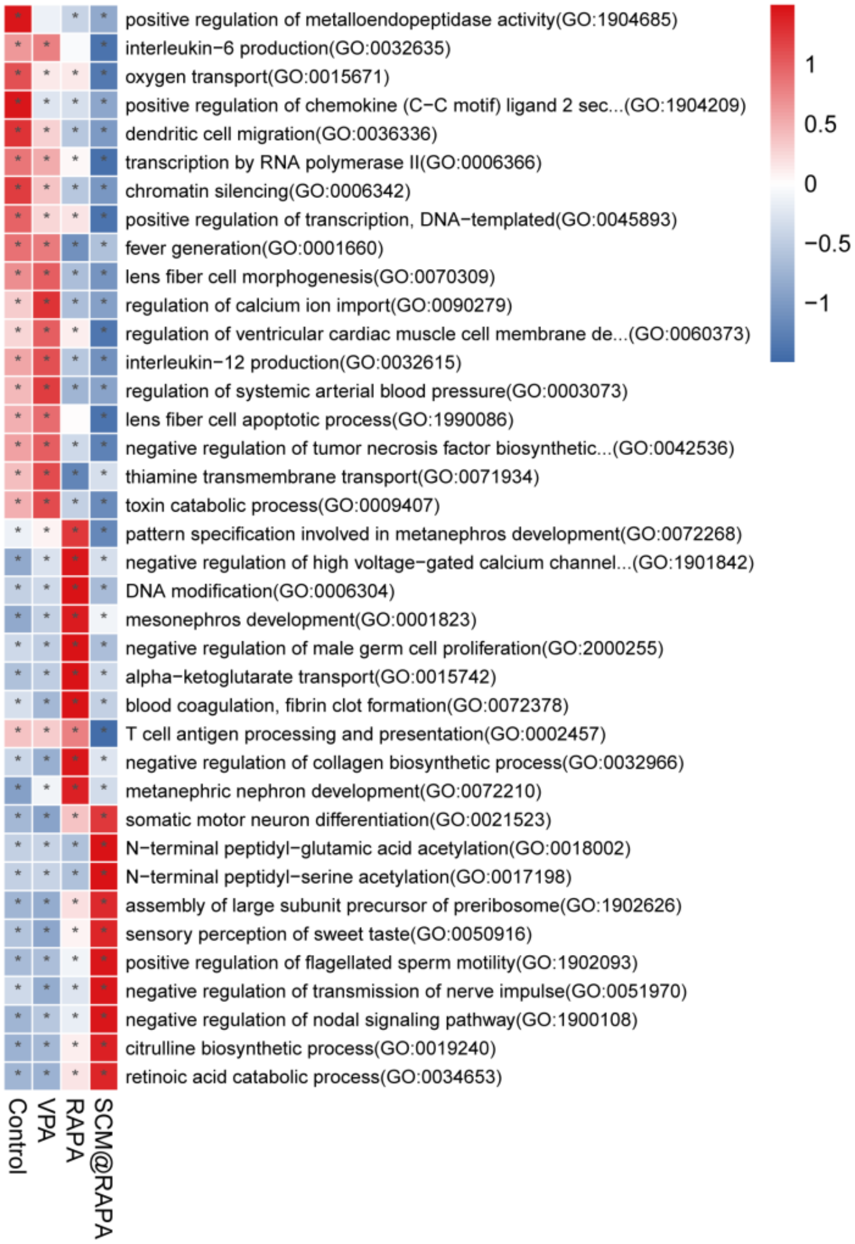

Figure S17. Heatmap of gene set variation analysis (GSVA) of top 10 biological process pathways in neurons.

Table S1. Statistical table of differentially expressed genes among different groups, $pvalue<0.05$ and $FoldChange>1.2$.

Case	Control	Up_diff	Down_diff	Total_diff
SCM@RAPA (group)	RAPA (group)	111	61	172
SCM@RAPA (group)	VPA (group)	154	102	256
RAPA (group)	VPA (group)	138	171	309
VPA (group)	Control (group)	33	82	115
